# Lower ototoxicity and absence of hidden hearing loss point to gentamicin C1a and apramycin as promising antibiotics for clinical use

**DOI:** 10.1038/s41598-019-38634-3

**Published:** 2019-02-20

**Authors:** Masaaki Ishikawa, Nadia García-Mateo, Alen Čusak, Iris López-Hernández, Marta Fernández-Martínez, Marcus Müller, Lukas Rüttiger, Wibke Singer, Hubert Löwenheim, Gregor Kosec, Štefan Fujs, Luis Martínez-Martínez, Thomas Schimmang, Hrvoje Petković, Marlies Knipper, M. Beatriz Durán-Alonso

**Affiliations:** 1 Molecular Physiology of Hearing, Department of Otolaryngology, Tübingen Hearing Research Centre (THRC), University of Tübingen, Tübingen, Germany; 2 Institute of Biology and Molecular Genetics (IBGM), University of Valladolid-CSIC, Valladolid, Spain; 3 Acies Bio d.o.o., Ljubljana, Slovenia; 4 University Hospital Marqués de Valdecilla IDIVAL, Santander, Spain; 5 Universidad de Cantabria, Santander, Spain; 6 Department of Otorhinolaryngology, Tübingen Hearing Research Centre (THRC), Regenerative Medicine, University of Tübingen, Tübingen, Germany; 7 Unit of Microbiology, University Hospital Reina Sofía, Córdoba, Spain; 8 Instituto Maimónides de Investigación Biomédica de Córdoba (IMIBIC), Córdoba, Spain; 9 Department of Microbiology, University of Córdoba, Córdoba, Spain; 10 Biotechnical Faculty, University of Ljubljana, Ljubljana, Slovenia; 11 Graduate School of Medicine, Department of Otolaryngology, Kyoto University, Kyoto, Japan

## Abstract

Spread of antimicrobial resistance and shortage of novel antibiotics have led to an urgent need for new antibacterials. Although aminoglycoside antibiotics (AGs) are very potent anti-infectives, their use is largely restricted due to serious side-effects, mainly nephrotoxicity and ototoxicity. We evaluated the ototoxicity of various AGs selected from a larger set of AGs on the basis of their strong antibacterial activities against multidrug-resistant clinical isolates of the ESKAPE panel: gentamicin, gentamicin C1a, apramycin, paromomycin and neomycin. Following local round window application, dose-dependent effects of AGs on outer hair cell survival and compound action potentials showed gentamicin C1a and apramycin as the least toxic. Strikingly, although no changes were observed in compound action potential thresholds and outer hair cell survival following treatment with low concentrations of neomycin, gentamicin and paromomycin, the number of inner hair cell synaptic ribbons and the compound action potential amplitudes were reduced. This indication of hidden hearing loss was not observed with gentamicin C1a or apramycin at such concentrations. These findings identify the inner hair cells as the most vulnerable element to AG treatment, indicating that gentamicin C1a and apramycin are promising bases for the development of clinically useful antibiotics.

## Introduction

Currently, there is a critical shortage of effective antibiotics, and in particular, those needed to treat serious infections caused by Gram-negative pathogens belonging to the *Enterococcus faecium*, *Staphylococcus aureus*, *Klebsiella pneumoniae*, *Acinetobacter baumannii*, *Pseudomonas aeruginosa* and *Enterobacter* species (ESKAPE) group. These include carbapenemase-producing enterobacteria, multidrug-resistant (MDR) *Pseudomonas aeruginosa* and MDR *Acinetobacter baumannii*^1,2^, which are the pathogens that pose the highest risk in clinical practice. Although aminoglycoside antibiotics (AGs) show potent antimicrobial activity, important side-effects have severely curtailed their use, such as nephrotoxicity, loss of vestibular function (i.e., vestibulotoxicity) and permanent hearing impairment (i.e., cochleotoxicity)^3,4^. In the 1970’s, AGs were mostly replaced by other antibiotics that are safer and highly efficacious against Gram-negative infections, such as the fluoroquinolones. However, in recent years the rapid spread of fluoroquinolone-resistance has seriously reduced the efficacy of these compounds in the fight against severe bacterial infections, such as tuberculosis, pneumonia, peritonitis, bacteremia, and intra-abdominal and genitourinary tract infections^5^. The current rise in MDR pathogens has highlighted the important role AGs have as the ultimate weapons against life-threatening infections^6–8^. It is therefore of great importance to identify AGs that have strong antibacterial activity while lacking their most harmful side-effects.

We carried out an extensive study on the ototoxic activities of five selected natural AGs^9^ following their confirmation as potent antibiotics against a selection of MDR clinical isolates of the ESKAPE panel: Neomycin (Neo), gentamicin (GM), paromomycin (Paro), apramycin (Apra) and gentamicin C1a (GM C1a). Neo, GM and Paromomycin are AGs that are medically used (e.g., https://www.drugs.com/monograph/gentamicin-sulfate.html)^10–12^ despite their known ototoxicity. Their antibacterial activities were compared to those of apramycin and GM C1a (congener present in the commercial GM mixture). Although apramycin is an antibiotic that is currently used in veterinary medicine^13^, neither apramycin nor GM C1a have been used in the clinic. However, a few studies have indicated that apramycin and GM C1a are antibacterials with lower ototoxic activity^14–16^.

Our *in-vitro* and *in-vivo* assays indicated dose-dependent toxicity of each of the AGs tested. When their ototoxicity was examined *in-vivo*, there was no hair cell (HC) loss or compound action potential (CAP) threshold shifts 3 weeks after a single application of AGs to the round window membrane at the lowest concentration under study. However, for neomycin, GM and paromomycin, but not apramycin or GM C1a, this concentration induced a reduction in inner hair cell (IHC) ribbon numbers (i.e., IHC synaptopathy), and a worsening of the CAP amplitudes. At higher concentrations, the AGs additionally induced a reduction in summating potential (SP) amplitudes, which mainly represented mechanoelectrical transduction (MET) current-mediated IHC receptor potential responses from basal cochlear turns. Importantly, these data thereby demonstrated hidden hearing loss caused by neomycin, GM and paromomycin but not apramycin or GM C1a, at concentrations where outer hair cell (OHC) loss was not yet detectable. Thus the IHC was identified as the most vulnerable element of the inner ear to AG treatment, and the data indicated IHC synaptopathy as the earliest marker of AG-induced ototoxicity. These data suggest that current GM administration regimes adopted in the clinic (i.e., application of GM at reduced doses or at once-a-day intervals) to reduce the risk of nephrotoxicity and ototoxicity might still cause hidden hearing loss. Thus, this might translate into hearing impairment later in life, as has already been observed following noise exposure^17^. On the other hand, these results underline the potential of apramycin and GM C1a as promising drug leads for the development of safer AGs for the clinic.

## Results

### Antibacterial efficacies of the selected aminoglycoside antibiotics

The antibacterial activities of GM, GM C1a, apramycin, paromomycin and neomycin were evaluated, and their minimum inhibitory concentrations (MICs) are given in Table 1 and Supplementary Table S1.

**Table 1:** Activities of the selected aminoglycosides against the clinical isolates of the five bacterial species.

Isolate	Gentamicin			Gentamicin C1a			Apramycin			Paromomycin			Neomycin		
	MIC		Range	MIC		Range	MIC		Range	MIC		Range	MIC		Range
	50	90		50	90		50	90		50	90		50	90	
Eco	2	>64	≤0.5–>64	1	>64	1–>64	8	16	4–32	4	>64	≤0.5–>64	1	>64	≤0.5–>64
Kpn	16	>64	≤0.5–>64	>64	>64	1–>64	4	>64	2–>64	2	>64	≤0.5–>64	1	>64	≤0.5–>64
Pae	8	>64	1–>64	1	>64	≤0.5–>64	8	>64	4–>64	>64	>64	4–>64	16	>64	≤0.5–>64
Ab	>64	>64	≤0.5–>64	16	>64	2–>64	16	64	2–>64	32	>64	1–>64	8	>64	1–>64
Sau	>64	>64	≤0.5–>64	1	32	2–>64	16	16	4–16	>64	>64	≤0.5–>64	>64	>64	≤0.5–>64

MIC_50_ and MIC_90_ values of both GM and the GM C1a congener were similar, with only a 2-fold increase in the MIC_50_ of GM C1a over that of GM. Importantly, this was not due to these compounds having the same or similar MIC values against exactly the same test organisms (shown in Supplementary Table S2). Good *in-vitro* activity of apramycin was recorded against MDR organisms, including carbapenem-resistant enterobacteria, and within a narrower range than GM or GM C1a. Eco, *Escherichia coli*; Kpn, *K. pneumoniae*; Pae, *P. aeruginosa*; Ab, *A. baumannii*; Sau, *S. aureus*.

Isolation of the GM C1a congener was achieved following derivatization of the GM C complex in the GM mixture using benzyl chloroformate, which provided a mixture of carboxybenzyl (Cbz)-protected GM congeners that were detected and separated using preparative HPLC with UV detection (Supplementary Fig. S1; for details, see Supplementary Information). A Cbz-protection / deprotection strategy was subsequently applied (Supplementary Fig. S2; for details, see Supplementary Information) that yielded gram quantities of pure GM C1a in the form of a disulfate salt (Supplementary Fig. S3; for details, see Supplementary Information).

The antibiotic activities of the selected AGs were established against an ESKAPE panel of 61 MDR isolates (Table 1, Supplementary Table S1). High MIC_90_ values (>64 mg/L) were obtained for almost all of these compounds, with the exception of apramycin (MIC_90_, 32 mg/L). However, in terms of MIC_50_, the most active compounds were neomycin and paromomycin. Interestingly, although the MIC_50_ and MIC_90_ values of GM and GM C1a were similar, these two compounds showed different *in-vitro* activities against a large proportion of the isolates (Supplementary Table S2). The MICs determined for GM were within the defined quality control ranges for the three reference strains, as defined by the Clinical and Laboratory Standards Institute (CLSI, 2018). As the breakpoints for defining clinical categories are only established for GM, by the CLSI and the European Committee on Antimicrobial Susceptibility Testing, it is difficult at present to evaluate the clinical value of the information obtained for the other tested compounds, such as GM C1a and apramycin. However, if the *in-vitro* activity of GM C1a is evaluated considering the same breakpoints defined for GM, then 42.6% (26/61) and 6.6% (4/61) of the isolates tested would be susceptible (<8 mg/L) and intermediate resistant (8 mg/L), respectively, to GM C1a. Categorical breakpoints have been suggested for apramycin^18^ based on information from a National Antibiotic Resistance Monitoring Study report, and its susceptible, intermediate resistant, and resistant categories are defined at ≤8 mg/L, 16–32 mg/L, and ≥64 mg/L, respectively. Considering these breakpoints, 59.0% (36/61) and 31.1% (19/61) of the tested organisms would be susceptible and intermediate susceptible to apramycin, respectively (Table 1). Additionally, the MICs for apramycin were within the narrow range of 4 mg/mL to 16 mg/L for 82.0% (50/61) isolates, whereas only three isolates (*K. pneumonia*e [×2], *A. baumannii*) had MICs > 64 mg/L (Table 1, Supplementary Table S1).

### Toxicity to otic cell lines varies among aminoglycoside antibiotics

*In-vitro* evaluation of the ototoxicities of neomycin, GM, paromomycin, apramycin and GM C1a was conducted on two immortalized otic cell lines (House Ear Institute-Organ of Corti 1 [HEI-OC1], OC-k3 cells), both derived from the organ of Corti of postnatal day 14 (P14) H-2Kb-tsA58 transgenic mice^19–21^. MTT tests (using 3-[4,5-dimethylthiazol-2-yl]-2,5-diphenyltetrazolium bromide) were used to detect metabolically active cells in the cultures. Initial experiments were carried out with the ototoxic AGs GM and neomycin, to establish the optimal conditions for conducting these tests. Three AG concentrations (1, 2, 5 mM) were analyzed for three different incubation times (24, 48, 72 h). At 5 mM, both GM and neomycin were toxic to both cell lines following the 24-h treatment (Supplementary Fig. S4a). Application of 2 mM GM or neomycin for 48 hours resulted in a statistically significant loss of viability in the cultures, albeit this was significantly lower than for 5 mM GM or neomycin (Supplementary Fig. S4b). Overall, no increased toxicity was seen for the 72-h treatment with 5 mM GM or neomycin, compared to 48 h. On the other hand, 1 mM and 2 mM GM or neomycin for 24 h showed no significant toxicities for either cell line (Supplementary Fig. S4a). Therefore, *in-vitro* ototoxicity was thereafter evaluated by conducting MTT tests on cultures treated for 48 h with 2 and 5 mM AGs.

**Figure 1:**
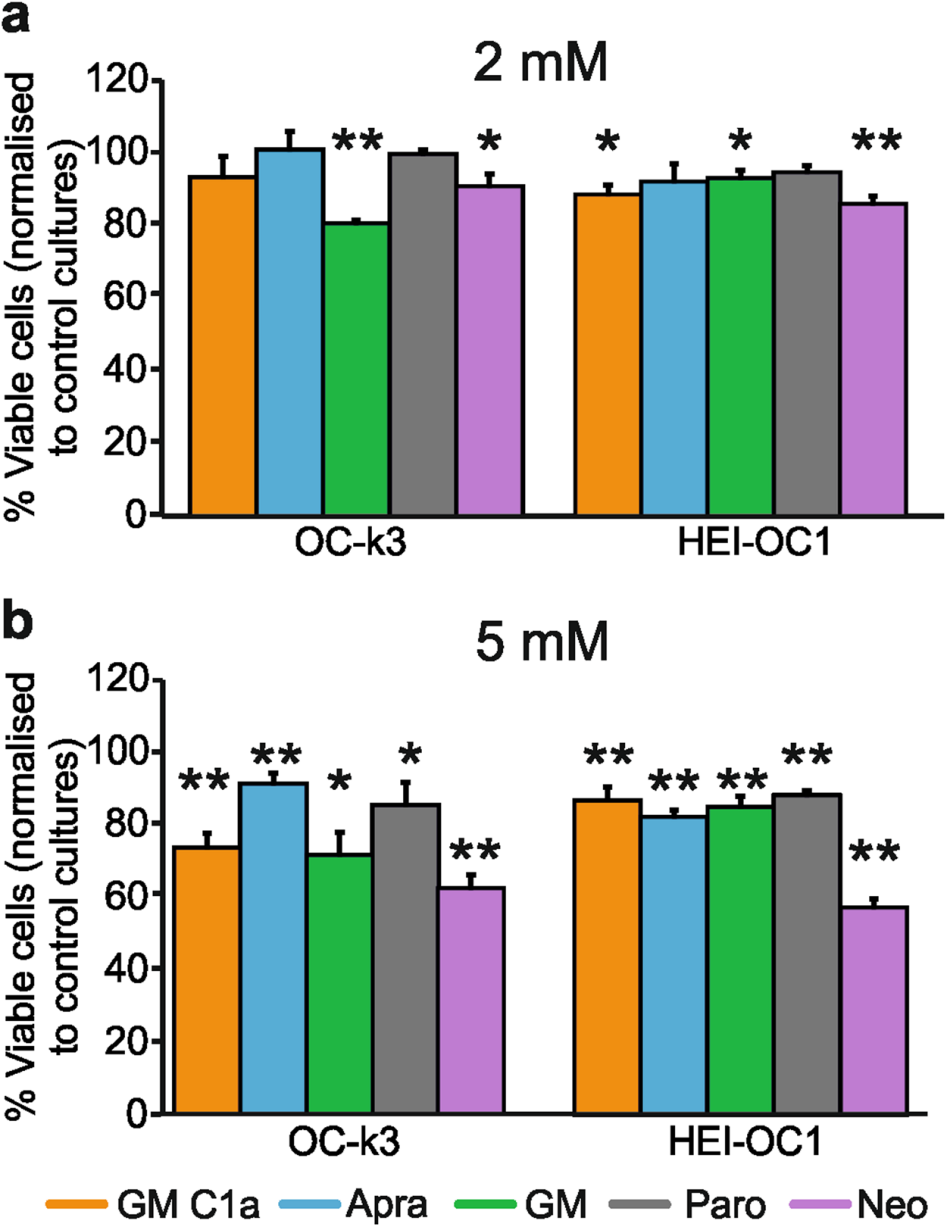
Viability of OC-k3 and HEI-OC1 cell cultures following AG treatments. Selected AGs were applied to the OC-k3 and HEI-OC1 otic cell lines at 2 mM (**a**) and 5 mM (**b**). After 48-h, the viability of the cells were determined using the MTT test, comparing the treated cells to the control cells (100%). Data are presented as means ± SD. *p < 0.05; **p < 0.01 (two-tailed unpaired Student’s t-tests used to compare each treatment to controls). (**a**) 2 mM AGs, OC-k3 cells: GM C1a, *p* = 0.3; Apra, *p* = 0.87; GM, *p* = 0.00000; Paro, *p* = 0.8; Neo, *p* = 0.016; (**a**) 2 mM AGs, HEI-OC1 cells: GM C1a, *p* = 0.01; Apra, *p* = 0.18; GM, *p* = 0.016; Paro, *p* = 0.051; Neo, *p* = 1.99E-5; (**b**) 5 mM AGs, OC-k3 cells: GM C1a, *p* = 0.003; Apra, *p* = 0.009; GM, *p* = 0.01; Paro, *p* = 0.043; Neo, *p* = 0.0005; (**b**) 5 mM AGs, HEI-OC1 cells: GM C1a, *p* = 0.006; Apra, *p* = 3.3E-6; GM, *p* = 0.001; Paro, *p* = 0.0001; Neo, *p* = 5.5E-5). Abbreviations: AG, aminoglycoside; Apra, apramycin; GM, gentamicin; GM C1a, gentamicin C1a; MTT, 3-[4,5-dimethylthiazol-2-yl]-2,5-diphenyltetrazolium bromide; Neo, neomycin; Paro, paromomycin.

At 2 mM AG concentrations, only GM and neomycin were confirmed as toxic to both cell lines (Fig. 1a). A trend toward greater survival that did not reach statistical significance was seen for OC-k3 cells treated with 2 mM GM C1a (93.3% ± 5.9%; no significant loss of viability compared to controls; *p* > 0.1) compared to 2 mM GM (80.7% ± 0.8%; significant loss of viability compared to controls; *p* < 0.0001) (Fig. 1a). This difference was not seen for HEI-OC1 cells, where 2 mM GM C1a showed significant toxicity which was similar to that of 2 mM GM or neomycin (Fig. 1a). Paromomycin and apramycin at 2 mM did not show any toxicity toward either of these cell lines, with significantly higher survival for OC-k3 cells treated with 2 mM apramycin, compared to 2 mM GM.

Treatment of both of the cell lines with all of the AGs at 5 mM led to significant loss of viability (Fig. 1b), with neomycin as the most toxic. Interestingly, the cells showed differences in their responses to some of the AGs. The HEI-OC1 cells were significantly more resistant to GM and GM C1a than the OC-k3 cells, while they were more susceptible to apramycin. On OC-k3 cells apramycin was significantly less toxic than neomycin, GM, and GM C1a.

Thus, these *in-vitro* ototoxicity tests indicated that neomycin was the most toxic of these AGs, with apramycin and paromomycin showing lower ototoxicities. In addition, for the OC-k3 line, 2 mM GM C1a appeared to be less toxic than 2 mM GM. Overall, the responses of both of these cell lines to the AGs were comparable, although the HEI-OC1 cells were significantly more resistant to 5 mM GM and GM C1a, and more sensitive to 5 mM apramycin, compared to the OC-k3 cells.

### Cochleotoxicity on organotypic cultures of cochlear epithelia varies among aminoglycoside antibiotics

**Figure 2:**
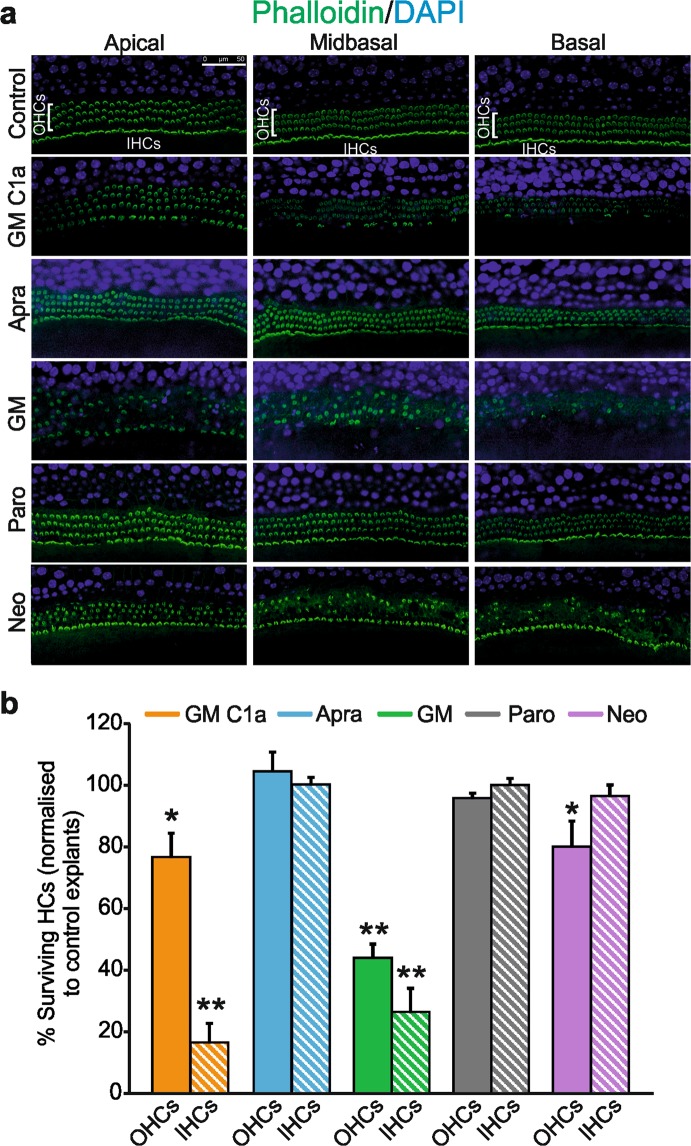
Evaluation of AG-induced ototoxicity on organotypic cultures of cochlear epithelia. (**a**) Explant cultures were treated with 0.1 mM AG for 23 h, and surviving HCs were detected following immunostaining of their stereocillia with phalloidin (a; from left to right: apical, middle, basal zones). Scale bar, 50 µm. (**b**) Proportions of surviving OHCs (full bars) and IHCs (striped bars) in AG-treated explants, relative to controls (100%). Data are presented as means ± SD. *p < 0.05; **p < 0.01 (two-tailed unpaired Student’s t-tests used to compare numbers of surviving HCs in each treatment group, to those in untreated controls). OHC survival: GM C1a, *p* = 0.01; Apra, *p* = 0.52; GM, *p* = 3.7E-8; Paro, *p* = 0.06; Neo, *p* = 0.044; IHC survival: GM C1a, *p* = 1.44E-8; Apra, *p* = 0.92; GM, *p* = 5.49E-7; Paro, *p* = 0.98; Neo, *p* = 0.36). A clear reduction in the numbers of surviving IHCs was observed in explants treated with GM and GM C1a. Here, IHC loss was similar for both AGs (26.5% ± 7.7%; 16.5% ± 6.3%; of surviving IHCs following GM and GM C1a treatments, respectively (*p* > 0.05)). Abbreviations: AG, aminoglycoside; Apra, apramycin; GM, gentamicin; GM C1a, gentamicin C1a; HC, hair cell; IHC, inner hair cell; Neo, neomycin; OHC, outer hair cell; Paro, paromomycin.

These AGs were tested on organotypic cultures of cochlear epithelia obtained from new born (P3) mice. Cochlear explants were treated with 0.1 mM AGs for 23 h (Fig. 2), conditions under which OHC survival was ~50% following GM treatment (see Supplementary Information). As reported previously^22,23^, a gradient was observed in the vulnerability of HCs to AG toxicity, with the HCs in the basal area of the cochlear epithelium more susceptible to damage than those in the apical zone (Fig. 2a). Treatment with apramycin showed no HC toxicity (104.5% ± 6.3% OHC survival), as for paromomycin (95.9% ± 1.6% OHC survival). In contrast, significant HC toxicity was seen for GM C1a (76.7% ± 7.7% OHC survival; *p* < 0.05), GM (43.9% ± 4.6% OHC survival; *p* < 0.001), and neomycin (80.0% ± 8.4% OHC survival; *p* < 0.05) (Fig. 2b). GM was significantly more toxic than the other AGs, including the GM C1a congener (*p* < 0.01). Interestingly, an apical-to-basal gradient of IHC loss was consistently observed for both GM and GM C1a (Fig. 2a), which indicated greater vulnerability of IHCs compared to OHCs. The same effects on the explants were seen for lower concentrations of GM and GM C1a (0.05 mM), and the testing of a different commercial GM mixture yielded the same results.

In summary, this study on organotypic cultures of cochlear epithelia indicated the toxicities of GM C1a, GM and neomycin. Although GM C1a was toxic to the explants, it was significantly less toxic than the commercial GM mixture. Indeed, GM was the most toxic of the AGs, with no toxicities seen for organotypic cultures treated with either apramycin or paromomycin.

### Dose-dependent elevation of compound action potential threshold shifts in the high frequency region varies among aminoglycoside antibiotics

Following the *in-vitro* studies, and to analyse the primary long-term ototoxic effects, we investigated the cochleotoxicity of these five AGs in an *in-vivo* guinea pig model, 3 weeks after application of each AG to the round window membrane. A hydrogel-based delivery system to the cochlea was used, which excluded indirect retrocochlear effects that might be caused by nephrotoxicity, for example, as described by Muller *et al*.^24^. These hydrogels have been successfully used in animal research and in the clinic, and they promote long-lasting effects^25^. Three defined AG concentrations were chosen (60 mg/mL, 210 mg/mL, 420 mg/mL) based on dose-response curves for neomycin and GM on CAP hearing thresholds. For this selected concentration range, none of the typical correlates of impaired vestibulo-ocular reflex, such as head tremble, head deviation, spontaneous nystagmus, eye deviation, body tilt or looping (not shown) were observed^26^ for any of the initial tests, not even at the highest AG concentrations tested. Impaired vestibulo-ocular reflex can result in loss of appetite and weight, which can lead to the death of the experimental animal. With the administration method used here, all 45 AG-treated animals survived. These observations confirm the advantage of local round window application in terms of a safe treatment, as it provides an animal model of AG-induced hearing loss with low mortality rates^27,28^.

**Figure 3:**
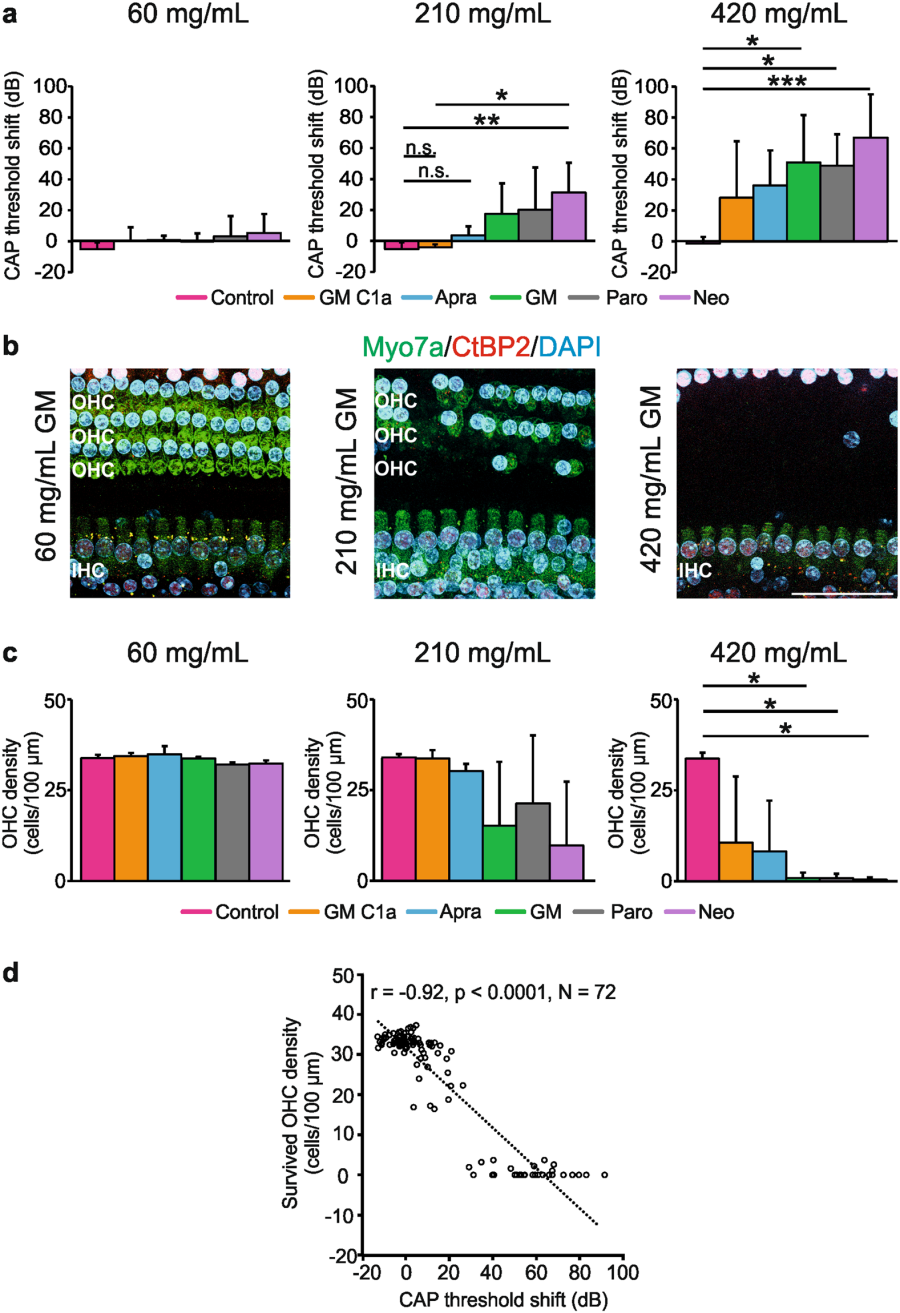
AG-induced cochleotoxicity on CAP threshold and OHC survival. (**a**) Comparison of CAP threshold shifts for the ‘basal region’ on day 21, in 60, 210 and 420 mg/mL groups. (**b**) Representative immunohistochemistry images of the basal cochlear turn showing HC loss following AG application, on day 21. Scale bar, 40 μm. (**c**) Comparison of surviving OHCs among six groups at three AG concentrations, on day 21. Surviving OHCs were counted in whole mounts of the basal cochlear turn along the basilar membrane. Data are shown as mean number of surviving OHCs per 100 µm distance of basilar membrane. (**d**) Analysis of the correlation between CAP threshold and OHC survival. X-axis, number of surviving OHCs per 10 µm distance of basilar membrane; Y-axis, CAP threshold shift (dB). The data are means ± SD. *p < 0.05; **p < 0.01; ***p < 0.001 (one-way ANOVA with Tukey’s multiple comparison tests). (a) 60 mg/mL *p* = 0.2082; 210 mg/mL *p* = 0.0044, 420 mg/mL *p* = 0.0024; (c) 60 mg/mL *p* = 0.2842, 210 mg/mL *p* = 0.1123, 420 mg/mL *p* = 0.0086. Abbreviations: AG, aminoglycoside; Apra, apramycin; CAP, compound action potential; GM, gentamicin; GM C1a, gentamicin C1a; IHC, inner hair cell; Neo, neomycin; O or OHC, outer hair cell; Paro, paromomycin.

The CAP measurements represent the summed response of the synchronous firing of all of the auditory nerve fibres (with lower or higher thresholds, and spontaneous firing rates)^29,30^. This allowed the extraction of direct response changes to sound within the cochlea following the AG application. To evaluate AG-induced cochleotoxicity on hearing function over time, the CAP threshold shifts were calculated by subtracting the CAP threshold before AG application from that after AG application. The CAP threshold shifts were analysed for round window application of the selected AG concentrations (60, 210 [data not shown], 420 mg/mL) after 60−90 min and for days 7, 14, and 21 (Supplementary Fig. S5a). All of the AGs showed dose-dependent CAP threshold increases, with the maximum values seen at day 7. The threshold shift differences were mainly at >4 kHz (i.e., corresponding to alterations in the ‘basal region’) in comparison to the measurements recorded at <4 kHz (‘mid region’). The CAP threshold shift between 60 min and 7 days also showed rapid onset and long-lasting elution times of the AGs from the hydrogel. The threshold shifts showed no changes from day 7 onwards at any of the frequencies (Supplementary Fig. S5a). The input-output function curves at day 21 showed that the AG-induced effects were mainly restricted to the ‘basal region’ (Supplementary Fig. S5a). The fine structure analysis of the CAP thresholds and amplitudes was completed with the data collected at the ‘basal region’ at both times (before application, day 21 following treatment), which indicated that 60 mg/mL, 210 mg/mL, and 420 mg/mL AGs induced dose-dependent increases in the CAP thresholds (Supplementary Fig. S5b; n = 3 animals/6 ears per AG). The CAP threshold differences for each AG 3 weeks after the round window application are illustrated in Fig. 3. In comparison to the vehicle-treated groups, application of 420 mg/mL of any of the AGs tested resulted in significant CAP threshold shifts (Fig. 3a; n = 3 animals/6 ears; *p* = 0.0024; GM C1a, *p* = 0.049; Apra, *p* = 0.001; GM, *p* = 0.0001; Paro, *p* = 0.016; Neo, *p* = 0.0005). When the AGs were applied at 210 mg/mL, GM, paromomycin, and neomycin also led to significant CAP increases (Fig. 3a; n = 3 animals/6 ears, *p* = 0.004), while those for GM C1a and apramycin did not reach statistical significance (*p* > 0.05, with CAP threshold shifts <10 dB). No significant CAP threshold increases were seen with any of the AGs when they were tested at a 60 mg/mL (Fig. 3a; n = 3 animals/6 ears; *p* = 0.21).

As the thresholds for sound-evoked neural potentials are not affected by diffuse neuronal loss as long as the OHCs are functioning normally^31^, a normal CAP threshold is expected to reflect intact OHCs. Surface preparations of cochleae from guinea pigs treated with 420 mg/mL of all of the tested AGs, or with 210 mg/mL GM, paromomycin or neomycin, showed extensive OHC loss in the basal turns (see Fig. 3b). To quantify this effect, the surviving OHCs were counted in whole mounts of the basal cochlear turn along the basilar membrane. This decrease in OHC density was significant with 420 mg/mL AGs (Fig. 3c; n = 3 ears per AG; *p* = 0.0086), although the OHC loss for animals treated with 210 mg/mL GM, paromomycin or neomycin did not reach statistical significance (Fig. 3c; n = 3 ears per AG; *p* = 0.11). In agreement with the reduced CAP threshold shifts recorded at cochlear midbasal turns (Supplementary Fig. S5a), no significant OHC loss was observed in the ‘mid region’ (data not shown). When the animals were treated with 60 mg/mL AGs, there was also no OHC loss in the ‘basal region’ (Fig. 3c; n = 3 ears per AG; *p* = 0.28). In support of the robustness of these findings, there was a significant negative correlation between the CAP threshold shifts and the density of the surviving OHCs, as shown for the ‘basal’ and ‘mid regions’ (Fig. 3d; r = −0.92; *p* < 0.001; n = 72).

Taken together, dose-dependent increases in the CAP thresholds were seen that correlated with the loss of OHCs in the basal regions of the cochlea. Apramycin and GM C1a showed the weakest effects on cochlear function, while GM, paromomycin and neomycin showed the highest toxicities.

### Loss of compound action potential amplitude and synaptic ribbons varies among aminoglycoside antibiotics in the absence of compound action potential threshold shifts

**Figure 4:**
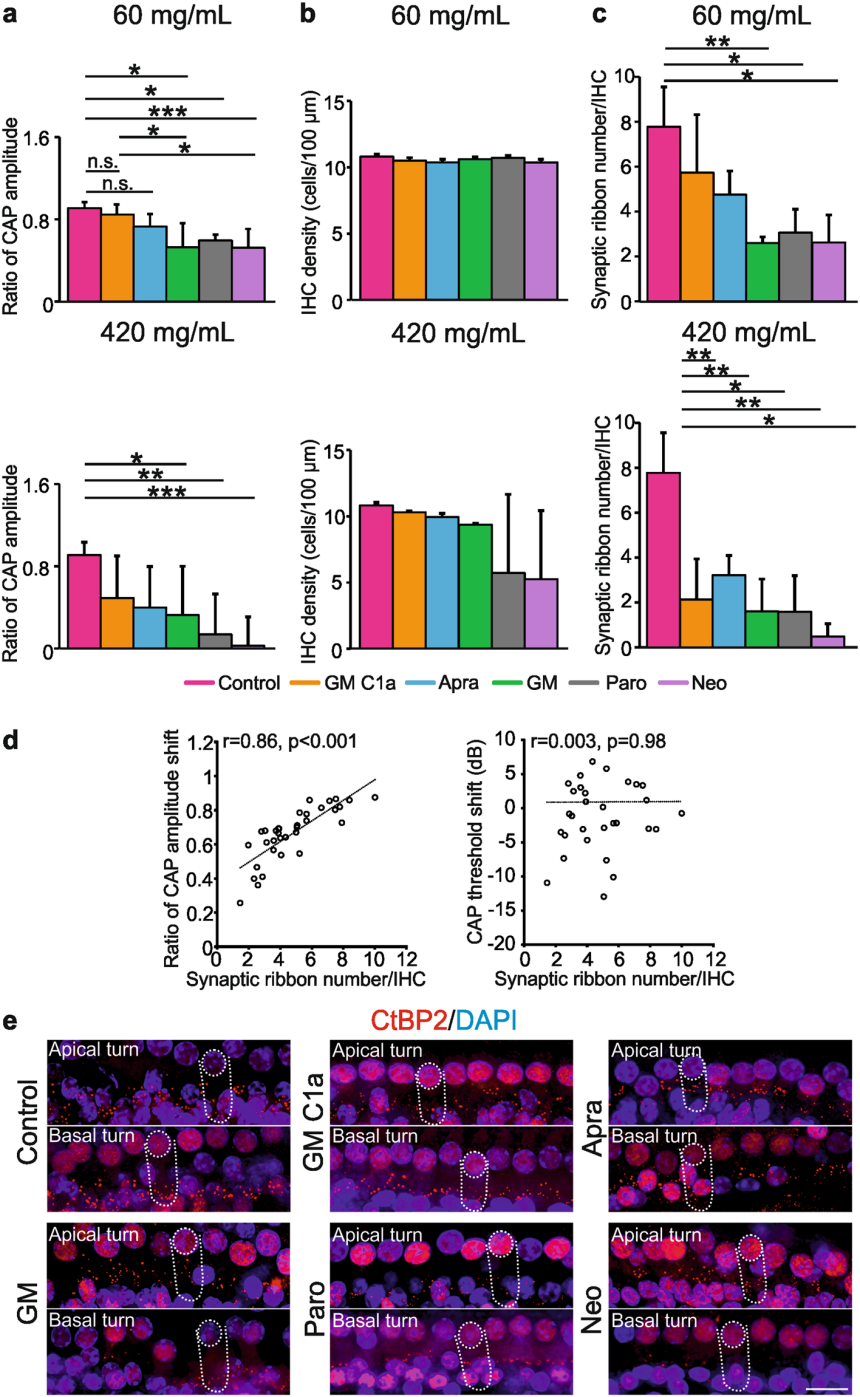
AG-induced cochleotoxicity on CAP amplitude and survival of IHC elements. (**a**) Comparison of ratios of CAP amplitudes (day 21/ before application) for the ‘basal region’ (60, 420 mg/mL). (**b**) Quantitative analysis of surviving IHCs, on day 21. (c) Survival of synaptic ribbons, on day 21, following administration of 60 or 420 mg/mL AGs. Data are means ± SD. *p < 0.05; **p < 0.01; ***p < 0.001 (one-way ANOVA with Tukey’s multiple comparison tests). (**a**) 60 mg/mL *p* < 0.0001, 420 mg/mL *p* = 0.0009; (**b**) 60 mg/mL *p* = 0.0764, 420 mg/mL *p* = 0.1194; (**c**) 60 mg/mL *p* = 0.0051, 420 mg/mL *p* = 0.0011). Correlations between the ratio of CAP amplitude (day 21/before application)/CAP threshold shifts and synaptic ribbon number in animals receiving 60 mg/mL AGs (n = 34) (**d**). Representative immunohistochemistry images showing synaptic ribbon loss in the IHCs after applying either control solution or AGs. (**e**) Scale bar, 20 µm. Abbreviations: AG, aminoglycoside; Apra, apramycin; CAP, compound action potential; CtBP2, C-terminal-binding protein 2; GM, gentamicin; GM C1a, gentamicin C1a; Neo, neomycin; Paro, paromomycin.

To get a more detailed view of the distinct cochleotoxic action of each individual AG, the CAP amplitudes at 21 days post-treatment were compared to those before application. These CAP input-output functions reveal the functional status of the auditory nerve fibres/synapses in more detail. When 210 mg/mL (not shown; *p* = 0.022) and 420 mg/mL AGs were applied, significant decreases in the CAP amplitude were seen (Fig. 4a; lower panel; n = 6 ears per AG; *p* = 0.0009). Interestingly, there was also a significant decrease of the CAP amplitude at the ‘basal region’ in animals treated with 60 mg/mL GM, paromomycin or neomycin (Fig. 4a; upper panel; n = 6 ears per AG; *p* < 0.0001), although at this AG concentration the CAP thresholds were unchanged compared to the controls. No decrease in the CAP amplitude was seen for 60 mg/mL GM C1a or apramycin.

Considering that neither CAP threshold shifts nor OHC loss were observed following the administration of 60 mg/mL of any of the AGs, the decline in CAP amplitude after treatment with either GM, paromomycin or neomycin was surprising, and this indicated decreased sound responsiveness at the level of the IHCs. Interestingly, no significant changes in IHC density were observed for any of the AG treatments, neither for the ‘mid regions’ (not shown) nor for the ‘basal regions’ (Fig. 4b; n = 3 ears per AG; *p* > 0.05), which indicated that the decreased CAP amplitude after the lowest AG treatments is not linked to IHC loss.

Various studies have indicated that the number of presynaptic ribbon-like transmitter release sites at the base of IHCs can be used as an approximate readout of IHC afferent innervation^32^. These sites can be stained with antibodies directed against CtBP2/ RIBEYE (see Fig. 4e for a whole-mount preparation)^32^. When the synaptic ribbon numbers were counted in whole-mount cochlear explants of AG-treated animals, significant IHC ribbon decrease was observed in the basal turn (Fig. 4c,e; n = 3 ears per AG/12–16 IHCs per ear) of the animals treated with AGs at 420 mg/mL (*p* = 0.0011) (Fig. 4c), and 210 mg/mL (*p* = 0.0279; data not shown). This was also the case for 60 mg/mL GM, paromomycin or neomycin, as shown by the *post-hoc* tests (Fig. 4c,e; n = 3 ears per AG; *p* = 0.0051). Importantly, these findings indicated that GM, paromomycin and neomycin, but not GM C1a or apramycin, can induce a loss of CAP amplitude and IHC synaptic ribbons at concentrations that do not result in any OHC loss or elevation of CAP thresholds. Indeed, when the CAP amplitude was compared with the synaptic ribbon number in basal cochlear turns, a strong correlation was found (Fig. 4d; left panel; r = 0.86; *p* < 0.001; n = 34), while CAP threshold and IHC ribbon numbers did not correlate (Fig. 4d, right panel). Therefore, the CAP amplitude appears to be an adequate metric for synaptopathy.

### Loss of summating potential amplitude, but not summating potential/compound action potential amplitude ratio, varies among aminoglycoside antibiotics in the absence of compound action potential threshold shifts

**Figure 5:**
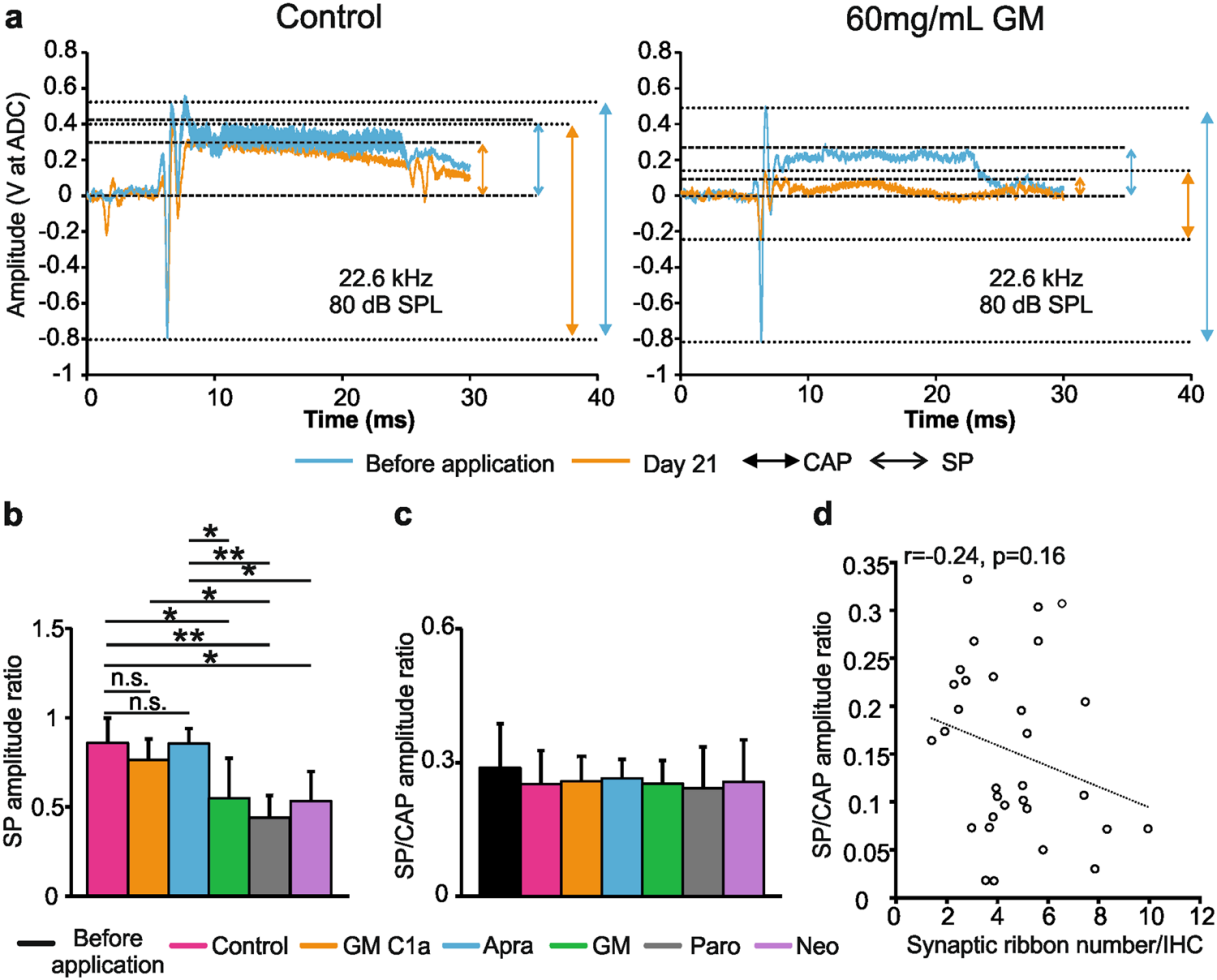
AG-induced effects on SP-, CAP- and SP/CAP amplitudes. (**a**) SP and CAP measurements in the control group (left) and the AG-treated group, before and 21 days after the application of 60 mg/mL GM (right), recorded at 80 dB sound pressure level of 22.6 kHz. (**b**) SP amplitude ratio among six groups on day 21. (**c**) SP/CAP amplitude ratio among seven groups (before application in all groups (n = 36), control and those receiving AGs). (**d**) Correlation between SP/ CAP amplitude ratio and synaptic ribbon number in the groups of animals treated with 60 mg/mL AGs (n = 32). Data are means ± SD. *p < 0.05; **p < 0.01; ***p < 0.001 (one-way ANOVA with Tukey’s multiple comparison tests). (**b**) *p* = 0.0001. (**c**) *p* = 0.846). Abbreviations: CAP, compound action potential; Apra, apramycin; GM, gentamicin; GM C1a, gentamicin C1a; Neo, neomycin; SP, Summating potential; Paro, paromomycin.

The detection of the SP reflects the direct current of receptor potentials. This applies mainly to that of IHCs^33,34^ in the basal cochlear turns^35,36^, as IHCs move in conjunction with the basilar membrane^29^. In contrast, OHCs in the base of the cochlea produce little direct-current receptor potential except at very high sound pressure levels^37–39^. SP responses thus provide a first indication of whether MET channel currents in the IHCs are intact or not^30,40^. On the other hand, enhanced SP/ action potential **(AP)** amplitude ratios (used as a measurement for CAP, and henceforth called the SP/CAP amplitude ratio) have been associated with IHC synaptopathy in cases such as auditory neuropathy due to mutations in the otoferlin gene^41^, and noise-induced hidden hearing loss^42^. On these bases, we analysed the SP amplitude and the SP/CAP amplitude ratios prior to AG application and at 21 days post-treatment (Fig. 5a, see Supplementary Fig. S5). At 21 days post-treatment with 60 mg/mL GM, paromomycin or neomycin, the SP amplitudes were significantly reduced compared to controls, GM C1a and apramycin (Fig. 5b; n = 6 ears per AG; *post-hoc* test *p* = 0.0001). Comparison of the SP/CAP amplitude ratios showed no significant differences among the two control groups and the five AG treatment groups (Fig. 5c; n = 6 ears per AG; *p* > 0.05). This indicated that GM, paromomycin and neomycin affect the SP amplitudes and CAP amplitudes in similar ways. In line with these data, the SP/CAP amplitude ratios were not influenced by the loss of synaptic ribbons (Fig. 5d; r = −0.24; *p = *0.16; n = 32).

In summary, all of the AGs except GM C1a and apramycin induced deterioration of SPs and reduction in synaptic ribbon numbers at concentrations that were well below the levels that affect the hearing (CAP) thresholds. The observation that the SP/CAP amplitude ratio did not change following treatment with GM, paromomycin or neomycin suggests that the AGs induce changes to the SP and CAP amplitudes to comparable extents.

## Discussion

The AGs remain a vital clinical asset, as they are very potent bactericidal compounds. Although their use is restricted due to severe side-effects (mainly nephrotoxicity and ototoxicity), the current rise in MDR bacterial infections makes it necessary to develop novel derivatives that have high antibacterial activities but have significantly lower toxicities. We evaluated the ototoxicities of five AGs after demonstrating their efficacies against a set of MDR pathogens from the ESKAPE panel.

We were particularly interested here to compare apramycin and GM C1a, as two AGs that have not yet been brought into the clinical setting, with neomycin and GM, two AGs with some medical indications that show good antibacterial activities, but with strong ototoxicities. This interest was motivated by our demonstration that GM C1a showed activity against a panel of ESKAPE MDR pathogens that was comparable to that of the commercial GM complex used in the clinic. In addition, we had also recorded very promising activity for apramycin (currently used as a veterinary antibiotic) against the panel of MDR pathogens. The fifth AG, paromomycin, was also included in these analyses in light of its high efficacy against the tested ESKAPE isolates.

Ototoxicity was evaluated using *in-vitro* and *in-vivo* models. The otic OC-k3 and HEI-OC1 cell lines^43–50^ were used as the *in-vitro* models, in addition to cultures of cochlear epithelia. Although derived from the same tissue and at the same developmental stage, it is possible that these cell lines differ regarding the cell types from which they were derived, as has already been described for similar cell lines^20^. As this might affect the way the cultures respond to treatments, parallel tests were carried out on both. Here, similar data were obtained, although the HEI-OC1 cells were significantly more resistant than the OC-k3 cells to treatments with 5 mM GM and GM C1a. Additionally, 2 mM AG treatment of OC-k3 cells, but not HEI-OC1 cells, indicated lower ototoxicity of GM C1a compared to GM, which was in agreement with the observations on the cochlear explants and in the *in-vivo* model. Surprisingly, the application of GM or GM C1a to cochlear organotypic cultures resulted in the loss of large proportions of IHCs. This loss was specific to GM and GM C1a throughout the various experimental settings, but was not seen in the *in-vivo* model. Differences have been observed among AGs in the mechanisms by which they induce HC loss^51^. Some studies have also shown that IHCs in cochlear explants are more vulnerable than OHCs to, e.g., salicylate treatment or oxygen-glucose deprivation^51,52^. However, this has not been described for AGs. As this specific sensitivity of IHCs to GMs was not seen in the *in-vivo* model here, at developmental stages that more closely resembled the clinical setting, no further work was conducted to investigate this in more detail. With regards to apramycin, the results obtained in all of these models indicated a lower toxicity of apramycin compared to the reference AGs neomycin and GM. Different data were obtained for the evaluation of the ototoxic potential of paromomycin. The cell lines and the cochlear explants indicated low toxicity for paromomycin, similar to that of apramycin, while the *in-vivo* studies demonstrated that it was clearly ototoxic, and comparable to neomycin and GM. Divergent data from *in-vitro* and *in-vivo* ototoxicity models have been recorded previously^53,54^. These might be related to the presence of vulnerable cell types *in-vivo* that have a role in the overall response to treatment and that are not present in *in-vitro* models, or to differential differentiation stages and signalling pathways being activated in the *in-vitro versus in-vivo* models^25,55–57^. Altogether, the *in-vivo* work here demonstrated lower cochleotoxicity of apramycin and GM C1a, compared to neomycin, GM, and paromomycin.

Application of the AGs is believed to initially cause stereociliary damage, which will be followed by HC loss, mainly of OHCs in the basal (high frequency) region^3^, which have been considered to date as the most vulnerable element of the inner ear to AG treatment. In addition, some studies have shown damage to the spiral ganglion neurons, which was thought to occur secondary to HC loss^58,59^. On the other hand, recent animal studies have shown that noise exposure can lead to spiral ganglion neuron degeneration, even when HCs recover and thresholds return to normal^32^. In noise-exposed ears showing no acute or chronic HC loss, a reduction of up to 50% of the synapses between IHCs and cochlear neurons can occur. The same primary loss of cochlear synapses occurs in the aging ear^17,60^. This cochlear synaptopathy has been termed “hidden” because cochlear neural degeneration does not cause behavioural changes or elevated electrophysiological thresholds until it becomes extreme^61,62^. Thus, the concept of “hidden hearing loss” refers to a disorder where a pure tone audiogram fails to detect any existing cochlear pathology and auditory processing deficits^32,63,64^. In these instances, although there is poor auditory speech recognition due to loss of cochlear synapses to IHCs and auditory nerve degeneration, normal hearing thresholds are maintained^64^.

Regarding this aspect, it is interesting that the IHC synaptic ribbons that form between IHCs and spiral ganglion neurons can be damaged following AG treatment, and independently from OHC loss. This feature adds support to previous speculation about IHCs being the primary vulnerable element to AG applications in the inner ear^65^. In our present *in-vivo* studies, the animals receiving 60 mg/mL neomycin, GM or paromomycin (and not those receiving 60 mg/mL apramycin or GM 1Ca) showed decreased numbers of IHC synaptic ribbons and reduced CAP amplitude in the absence of HC loss. These observations are interesting for several reasons:

(i) They confirm previously reported data that established a relationship between the amplitude of cochlear neural responses, such as auditory brainstem responses, and IHC synaptic ribbon loss, with both affected by aging^60^ and noise exposure^32^, and independent of hearing threshold loss^66^. We here used CAPs, which detect population responses of all of the auditory nerve fibers synchronously, which respond to an auditory stimulation of increasing sound pressure levels. The nature of this extracellular potential determines the response characteristic; i.e., more auditory fibers are activated with increasing sound pressure level. This leads to increase in the CAP amplitudes. Where fibers are not activated because their synaptic contact to the IHC is lost (e.g., in animal models of reduced ribbon counts or ribbon size in the presynapse^67^, using e.g. ouabain intoxication), a dose-dependent reduction in the size of the CAP amplitude is observed^67^. We can thus assume a direct association between the observed altered CAP amplitudes following AG treatment and the auditory nerve fiber contribution to the CAP response.
(ii) Our present *in-vivo* data demonstrate that animals that received 60 mg/mL neomycin, GM or paromomycin, but not those receiving 60 mg/mL apramycin or GM 1Ca, showed decreased SP amplitudes as a result of MET alterations, in parallel to decreased numbers of IHC ribbons and decreased CAP amplitude. As the SP/CAP amplitude ratio is not changed, both events may be related. When the AGs were applied at higher concentrations, they affected OHC survival with a strong decreasing gradient from the basal to the apical cochlear turns, which mainly means that OHCs in the basal cochlear turn are affected. In the basal cochlear turns (which represent higher frequencies) the SP is mainly generated by IHCs, at least at moderate intensities^37,39^. At higher frequencies, OHCs only contribute to the SP at high intensities (e.g., 110 dB sound pressure level). In the present study, the SP recordings were analyzed at 22.6 kHz and 80 dB sound pressure level, settings under which an OHC contribution should be minimal, or even absent.
(iii) Moreover, our data strongly support the concept that whether systemically or locally applied, AGs probably result in similar damage to the integrity of the IHC synapse, which will endanger IHC ribbon stability. Indeed, previous studies have shown IHC ribbon loss 2 weeks after an intraperitoneal injection of gentamicin^65^, which supports the concept that AGs can similarly affect IHC synapse integrity following systemic (intraperitoneal) or local application. In the absence of sufficient published data, we cannot however exclude that systemic administration of the lower concentrations of AGs might have yielded different results from the synaptopathy observed following the round window application; it is also hard to predict outcomes in human patients, which emphasizes the urgent need to attract industrial partners who can invest their efforts in the scaling up of isolation procedures, which is a step of great importance for patients in need of safer antibacterials.

For the mode of entry of AGs into HCs, two mechanisms have been proposed to date^68–70^: entry through the MET channels, which is regarded as the main route; and endocytosis. HC loss following AG entry appears to be mediated by mitochondrial dysfunction^16,71^, generation of reactive oxygen species^3^, and caspase activation^72,73^. It would thus be of interest to investigate whether the lower ototoxicity of apramycin and GM C1a might result from reduced affinity for MET channels, as has been demonstrated for some new sisomicin derivatives^6,16,69,74–76^. Indeed, although previous studies indicated ribbon loss following systemic treatment with 100 mg/kg GM, - loss that was not accompanied by any effects on stereocilia^77^-, we cannot exclude that structural damage to stereocilia rather than MET channel dysfunction affected SPs and CAP amplitudes following our AG administration protocol. This question can be addressed in future studies (e.g., using FM1-43X dye uptake assays).

In summary, this study has demonstrated the lower ototoxicity of the AGs apramycin and GM C1a, compared to those of the clinically used neomycin and GM. This translates into higher OHC survival and smaller changes in CAP thresholds following their application. Moreover, our studies have unveiled the potential of low concentrations of the AGs neomycin, GM and paromomycin to damage the IHC synapse and reduce CAP amplitudes preceding any damage to the OHCs. Gentamicin-induced loss of ribbon structures at the IHC has been observed before^77,78^. However, to date, no AGs have been differentially compared in terms of their potential to induce IHC synaptopathy, nor have auditory nerve functions been analysed to possibly link functional changes to damage to the IHC synapse. We observed reduced CAP amplitudes (but not CAP thresholds) linked to IHC ribbon loss upon AG treatment. The CAP amplitude changes might be reflected in supra-threshold ABR wave fine structures, which are currently not examined in routine clinical audiometry investigations. A decrease in SP amplitude following application of high AG concentrations was also indicative of the ongoing IHC dysfunction and altered mechanoelectrical transduction caused by AGs. While further studies are essential to analyse the effects of AGs on IHCs in more detail, our data point to the IHC as the primary target of AG treatment in the inner ear. The findings also emphasize the urgent need to consider the evaluation of supra-threshold ABR wave fine structure analysis as an approach for preclinical ototoxicity tests of AGs, as well as studying possible long-term ototoxic side effects of AGs in the clinic.

However, no IHC alterations were observed with apramycin or GM C1a, which further underlines the lower ototoxic potential of these compounds. Importantly, these AGs showed similar efficacies to neomycin and GM against a panel of ESKAPE pathogens. Based on these data, apramycin and GM C1a have potent antibacterial activities and low ototoxic potential, and thus they represent excellent starting points for chemical derivatization. Apramycin is already produced on an industrial scale as a veterinary product, and it is thus available in sufficient quantities. On the other hand, it is also highly relevant that the industrial production of individual AG congeners, such as GM C1a, can now be achieved following manual chemical derivatization^79^. Improvements in the biosynthetic processes for the production of individual components like GM C1a might therefore allow the industrial production of economically affordable antibiotics^80^. Although there are other pharmacological properties that apramycin and GM C1a will need to be evaluated for, such as their nephrotoxicity, the present data warrant further studies into these two AGs as promising candidates for translation into the clinical setting.

## Methods

### Aminoglycoside antibiotics and isolation of the congener GM C1a

Apramycin, GM and Neomycin were purchased from Glentham Life Sciences Ltd (UK); Paromomycin was purchased from AK Scientific Inc. (USA). Isolation of the GM C1a congener was carried out based on the procedure described by Grote and Johnson (2012)^79^. Benzyl chloroformate (Cbz)*-*protected derivatives of the GM complex were prepared so that they predominantly contained GM congeners of the C-series (see Supplementary Fig. S1), and these were separated by preparative HPLC (Supplementary Fig. S1). This strategy, followed by Cbz-deprotection (hydrogenation) and subsequent treatment with sulfuric acid, permitted the isolation of GM C1a in a pure form, as the sulfate salts (Supplementary Figs S2, S3). We established an efficient protocol for the isolation of gram quantities of the GM 1Ca congener, as described in the Supplementary Information.

For the evaluation of antibacterial activities and *in-vitro* ototoxicity testing, AGs were dissolved in distilled water at 50 mg/mL and sterilized by filtration. For *in-vivo* testing the AGs were prepared at three different concentrations (100, 350, 700 mg/mL) in demineralized water containing 1% polyethylene glycol (Sigma-Aldrich, St. Louis, MO, USA). Before surgery, each AG stock solution was diluted 3:2 in a 20% solution of Poloxamer 407 (Sigma-Aldrich, St. Louis, MO, USA), which provided 60, 210 and 420 mg/mL AG preparations in 1% polyethylene glycol and 20% Poloxamer 407^25^

### Antibacterial activities of the selected aminoglycoside antibiotics against multidrug-resistant clinical isolates

#### Clinical isolates used in this study

A collection of 61 MDR isolates (as previously defined Magiorakos *et al*.)^81^ were cultured from clinical samples (one isolate per patient) from the bacterial collection of the clinical microbiology laboratory of the University Hospital Marqués de Valdecilla (Santander, Spain). The organisms were identified with the Vitek-2 system (BioMérieux, France), following the manufacturer indications, and were conserved at −80 °C in tryptic soy broth with 10% glycerol. ESKAPE refers to a series of bacterial pathogens that are associated with antimicrobial resistance: *Enterococcus faecium*, *Staphylococcus aureus*, *Klebsiella pneumoniae*, *Acinetobacter baumannii*, *Pseudomonas aeruginosa*, and *Enterobacter* species. The following ESKAPE species with the indicated relevant resistance phenotypes were studied: 15 *Escherichia coli*, 10 of which produced extended-spectrum β-lactamases (ESBL), while 5 were not ESBL producers; 15 *K. pneumoniae*, 10 as ESBL producers, and 5 not ESBL producers; 11 *P. aeruginosa*, 7 as carbapenem resistant and 4 as carbapenem susceptible; 10 *A. baumannii*, as 5 carbapenem resistant and 5 as carbapenem susceptible; and 10 *S. aureus*, five as resistant to methicillin and five as susceptible to methicillin. *Enterococcus* spp. were not tested in this study, as these organisms are considered to be intrinsically resistant to AGs.

#### Antimicrobial susceptibility testing

Minimum inhibitory concentrations (MICs) of the five tested AGs were determined using in-house standardized broth microdilution, following CLSI guidelines (CLSI, 2012). Antimicrobial agents were tested in the range of 64 mg/L to 0.5 mg/L. After bacterial inoculation, the plates were incubated in air at 35 °C for 20 h. The MICs were read visually as the lowest concentration of AG inhibiting bacterial growth. *E. coli* ATCC 25922, *P. aeruginosa* ATCC 27853, and *S. aureus* ATCC 29213 were used as control strains. The MIC values calculated corresponded to concentrations that inhibited 50% (MIC_50_) and 90% (MIC_90_) of the 61 isolates.

### *In-vitro* ototoxicity testing

#### Cell cultures and viability tests

The auditory OC-k3 and HEI-OC1 cell lines were cultured according to Kalinec *et al*.^82^, in high-glucose Dulbecco’s modified Eagle’s medium (Gibco, Grand Island, NY, USA) supplemented with 2 mM glutamine and 10% fetal bovine serum (Gibco, Grand Island, NY, USA), and in the absence of antibiotics. The cells were passaged at a density of 2 × 10^5^ cells/mL and maintained under permissive conditions (i.e., 33 °C and 10% CO_2_, with addition of 50 U/mL interferon-γ (Sigma-Aldrich, St. Louis, MO, USA) to OC-k3 cultures).

When conducting MTT (Sigma Aldrich (St. Louis, MO, USA)) and ATP-based (Promega Corp, Madison, WI, USA) viability assays, the cells were plated onto 96-well flat-bottom plates (100 μL cell suspension/well) 24 h prior to AG treatment. Viability measurements were carried out following the manufacturer instruction.

#### Animals

The animal care, use and experimental protocols followed the national and institutional guidelines, and were reviewed and approved by the Animal Welfare Commissioner and the Regional Board of Animal Experimentation at the University of Tübingen and the University of Valladolid. All of the experiments were performed according to European Union Directive 2010/63/EU for the protection of animals used for experimental and other scientific purposes.

#### Organotypic cochlear cultures

Cochlear explants were prepared from postnatal day 3 (P3) C57/BL6 mice, according to the protocol described in Chen *et al*.^83^. Forty-eight hours after plating, they were treated with 0.1 mM AG for an additional 23 h, and subsequently fixed to stain the actin filaments in the stereocilia of the surviving HCs, with FITC-conjugated phalloidin (Molecular Probes Inc., Life Technologies, Eugene, OR, USA). Detailed information is provided in the Supplementary Information.

### *In-vivo* ototoxicity testing

#### Animals and experimental groups

Forty-eight adult guinea pigs that weighed between 350 and 600 g were used (pigmented, BFA bunt) and bred in an in-house animal facility, according to the German ‘Law on Protecting Animals’ (Tierschutzgesetz) and in line with European Directive 2010/63/EU for the protection of animals used for experimental purposes. Three animals were treated with each AG concentration (60, 210, 420 mg/mL). The AG solutions were applied bilaterally (i.e., six ears analysed per group).

#### Surgical procedures

All animals were anesthetized with an intramuscular injection of a mixture of fentanyl citrate (0.025 mg/kg; Fentadon, Eurovet Animal Health), midazolam (0.2 mg/kg; Midazolam-Ratiopharm, Ratiopharm) and medetomidine hydrochloride (Sedator, 1 mg/kg; EurovetAnimalHealth). Additional doses (20% of the initial dose) were applied as necessary after 2 h. During surgery and routine functional measurements, the animals were placed on a heating pad to maintain their temperature at 37 °C. An incision was made bilaterally in the post-auricular region to expose the otic bulla, and then a small hole was made in the bulla to identify the round window and the basal turn of the cochlea.

For functional measurements, an insulated 0.125-mm-diameter gold wire (Goodfellow, Cambridgeshire, UK) with a small hook at one end was placed at the ridge above the round window. Bilaterally implanted electrodes were connected to a percutaneous socket, and then fixed with dental cement to the vertex.

Once normal hearing was confirmed in each animal through functional measurements, the AG solution (10 μL) was delivered bilaterally to the round window membrane of the cochlea. A micromanipulator was used to place a 20 μL microloader pipette tip (Eppendorf, catalogue no. 5242-956.003) on the round window, which was connected to a 50 μL Hamilton syringe. The pipette tip was removed 15 min after the application, and then the bulla was sealed with dental acrylic, followed by suturing of the skin. Once the surgery and the functional measurements were completed, the anesthesia was antagonized using a subcutaneous (s.c.) injection of a mixture of Naloxan (0.03 mg/kg), Flumazenil (0.1 mg/kg), and Atipamezol (1 mg/kg). For three consecutive days following surgery, the animals were given Meloxicam (analgesic, 0.5 mg/kg, s.c) and Enrofloxacin (antibiotic, 10 mg/kg, oral).

#### Functional measurement and data collection procedures

Functional measurements were performed at five time points (before application, 60−90 min, days 7, 14, 21). Measurements of CAPs were carried out under anesthesia in a soundproof chamber, and performed as described previously^24^ (Supplementary Information).

#### Tissue preparation, immunohistochemistry, and hair cell and ribbon counts

The animals were euthanized 21 days after AG application, while under deep anesthesia using an overdose of Narcoren (4 mL/kg). Then, the cochlear samples were collected, preserved, permeabilized, and blocked, as described previously^84^. The cochlear tissues were stained to detect the CtBP2 (C-terminal-binding protein 2), as described in Supplementary Information.

### Statistical analyses

The *in-vitro* tests were each carried out in triplicate, for a minimum of three experiments (n = 3–8) carried out with each compound. All of the data are presented as means ± SD. Students’ t-tests were used to compare two groups, and two-way ANOVA with Holm-Sidak *post-hoc* analysis was used to compare three or more groups. The statistical software used was SigmaPlot v.11.0 (Systat). Values of *p* < 0.05 were considered to be statistically significant.

When evaluating the functional and histological data obtained from the *in-vivo* study, one-way ANOVA with Tukey’s multiple comparisons was used. To evaluate the correlation between the outcome variables and the histological findings, Pearsons correlations were used. All of the statistical analyses were performed using a commercially available software package (GraphPad Prism, GraphPad Software Inc., La Jolla, CA, USA). Values of *p* < 0.05 were considered to be statistically significant. All data are presented as means ± SD.

## Supplementary information

Supplementary methods, data and figures

## Data Availability

The datasets generated and/or analyzed during the current study are available from the corresponding authors upon request.
