# Supplementary material for: Lower ototoxicity and absence of hidden hearing loss point to gentamicin C1a and apramycin as promising antibiotics for clinical use
Source: Sci Rep. 2019 Feb 20;9:2410. doi: 10.1038/s41598-019-38634-3 (PMC6382871; doi:10.1038/s41598-019-38634-3)
Supplement: Supplementary file 1 — Supplementary methods, data and figures [file 41598_2019_38634_MOESM1_ESM.pdf]

Supplementary information

Lower ototoxicity and absence of hidden hearing loss point to gentamicin C1a and apramycin as promising antibiotics for clinical use

Masaaki Ishikawa[1], Nadia García-Mateo[2], Alen Čusak[3], Iris López-Hernández[2], Marta Fernández-Martínez[4,5], Marcus Müller[6], Lukas Rüttiger[1], Wibke Singer[1], Hubert Löwenheim[6], Gregor Kosec[3], Štefan Fujs[3], Luis Martínez-Martínez[7, 8, 9], Thomas Schimmang[2], Hrvoje Petkovic[3,10], Marlies Knipper[1]* and M. Beatriz Durán-Alonso[2]*

[1]Molecular Physiology of Hearing, Department of Otolaryngology, Tübingen Hearing Research Centre (THRC), University of Tübingen, Tübingen, Germany

[2] Institute of Biology and Molecular Genetics (IBGM), University of Valladolid-CSIC, Valladolid, Spain

[3]Acies Bio, d.o.o, Ljubljana, Slovenia

[4]University Hospital Marqués de Valdecilla IDIVAL, Santander, Spain

[5]Universidad de Cantabria, Santander, Spain

[6]Department of Otorhinolaryngology, Tübingen Hearing Research Centre (THRC), Regenerative Medicine, University of Tübingen, Tübingen, Germany

[7]Unit of Microbiology, University Hospital Reina Sofía, Córdoba, Spain.

[8]Instituto Maimónides de Investigación Biomédica de Córdoba (IMIBIC), Córdoba. Spain.

[9]Department of Microbiology, University of Córdoba, Córdoba, Spain.

[10]Biotechnical Faculty, University of Ljubljana, Ljubljana, Slovenia

*Corresponding authors

Methods

Isolation of the GM C1a congener.

Derivatization of the GM C sulphate complex with benzyl chloroformate (Cbz-Cl)

GM sulfate complex (Glentham Life Sciences, 5.00 g, 8.69 mmol, 1.0 equiv.) was dissolved in acetonitrile/water mixture (1:1, 100 mL) under basic conditions (Na_2CO_3, 9.21 g, 86.9 mmol, 10.0 equiv.). Benzyl chloroformate reagent (Cbz-Cl, 97 %, 9.94 g, 56.5 mmol, 6.5 equiv.) was added dropwise over 30 minutes. The reaction mixture allowed was stirred for 16 hours at ambient temperature (25 °C). After that period, the reaction mixture was partitioned between ethyl acetate and water. Phases were separated, and combined organic phases were washed with water (2x), saturated solution of ammonium chloride (1x), saturated solution of sodium chloride (1x) dried over Na_2SO_4, filtered and concentrated *in vacuum*. The crude pale-yellow oil (11.6 g) containing Cbz-protected GM congeners was further purified on silica gel using a flash column chromatography (Supplementary Fig. S1a).

Isolation of Cbz-GM C1a on the preparative HPLC

The crude material (11.6 g) was purified on silica gel eluting with dichloromethane (100%) to dichloromethane/methanol mixture (94:6). Fractions were collected into 30 mL glass test tubes. Based on the TLC analysis of column fractions, only the purest fractions were collected, combined and concentrated *in vacuum* which provided Cbz-protected GM congeners as white crispy solid (6.1 g, 61% yield). Subsequent LC/MS analysis revealed the presence of GM C1a in a form of a carbamate ester (Cbz-protected) eluting at t_R = 26.68 minutes (Supplementary Fig. S1b)

The separation of Cbz-protected GM C1a from a mixture of Cbz-protected GM C congeners was accomplished on Knauer preparative HPLC, with Smartline 2500 UV detector and 2 channel preparative pump 1800 utilizing VP150/32 Nucleodur C18 HTech 5 µm preparative column from Macherey-Nagel (Supplementary Fig. S1c). The preferred method included an isocratic elution. The mobile phase was composed of acetonitrile/water with 1‰ formic acid (60/40) and the method length was adjusted to 90 minutes. Loading capacity was between 300-400 mg of Cbz-protected GM per injection. Combined fractions of GM C congeners from

the column chromatography (previous step) were concentrated to dryness. The crispy residue (6.1 g) was dissolved in acetonitrile using an ultrasonic bath. However, the mixture was turbid due to poor solubility of Cbz-protected GM and it was centrifuged for 10 minutes at 4500 rpm. The collected supernatant (2 mL HPLC loop) was directly injected through the injector port on the preparative HPLC. The fractions were collected in the 15 mL centrifuge tubes and were further analysed by analytical HPLC instrument. From a single injection (400 mg of GM C/injection), it was possible to isolate approximately 80 to 100 mg of Cbz-GM C1a. Fifteen injections were required to purify the whole batch of Cbz-protected GM (6.1 g). According to the HPLC analysis, fractions containing the same Cbz-protected GM congener C1a were combined, concentrated and dried *in vacuo*. The Cbz-GM C1a congener purified with this protocol was analysed via LC/MS instrumentation. LC-MS analysis of isolated GM C1a is displayed in Supplementary Fig. S1d and S1e. The purified Cbz-GM C1a congener was isolated as white solid. Palladium-catalysed hydrogenation was utilized as a deprotection strategy for the effective removal of the Cbz group. We have demonstrated that the derivatization of the GM C complex with benzyl chloroformate provides a mixture of Cbz-protected GM congeners, which are easily detectable and separable using the preparative HPLC with UV detection. This strategy was successfully implemented for the expeditious purification and isolation of Cbz-GM C1a and other Cbz-GM congeners (Cbz-GM C2, Cbz-GM C2a and Cbz-GM C1) in gram quantities.

Pd-Catalyzed hydrogenation of pure Cbz-GM C1a congener

The Pd-catalyzed hydrogenation of Cbz-protected GM C1a congener was performed at ambient temperature (25 °C) and at 1 bar pressure of H_2 gas (Supplementary Fig. S2a and S2b). The vacuum dried Cbz-protected GM C1a (1.38 g, 1.23 mmol, 1.0 equiv.) was dissolved in methanol (50 mL). Palladium on carbon (10%, Pd/C, 213 mg/mmol of substrate, 261 mg) was added in a single portion, the flask evacuated and filled with hydrogen 3 times. The black suspension was stirred at ambient temperature, under 1 bar of H_2 pressure (balloon) for 16 hours. The suspension was filtered over celite filter aid, washed with methanol and

concentrated. The oily residue (GM C1a free base, 0.55 g, 1.23 mmol, 1.0 equiv.) was dissolved in distilled water (100 mL, pH = 10.05) and treated with 1.0 M H_2SO_4 (2.46 mL, 2.46 mmol, 2.0 equiv., pH = 3.5 - 4.0) to provide a disulphate salt, a more stable form of AG. Water was evaporated and the residue dried in high vacuum to provide GM C1a x $2H_2SO_4$ (0.79 g, 1.22 mmol, 99 % yield) in the form of crystalline powder. Pure GM C1a disulphate congener was analysed by LC/MS and it structure confirmed by ^{1}H- and ^{13}C-NMR spectroscopy (Supplementary Fig. S2c, S2d, S3a and S3b). In conclusion, we have successfully applied a Cbz-protection/deprotection strategy to prepare gram quantities of GM C1a in a pure form, as disulphate salt.

Analytical method used in the preparation of the GM C1a congener

The method required the use of a Thermo Acclaim RSLC PA2 100x2.1 mm HPLC column with a particle size of 2.2 μm. The mobile phase consisted of: 0.025% HFBA (hexafluoro butyric acid), 5% acetonitrile and 95% water (Component A), and 0.3% TFA (trifluoroacetic acid), 5% acetonitrile and 95% water (Component B). The mobile phase was pre-mixed in a ratio 1:1 and HPLC was operated at a mobile phase flow of 200 µl/min and column oven temperature of 60°C. The ESI probe parameters are described in Supplementary Table S3.

***In vitro* ototoxicity tests on organotypic cochlear cultures**

Inner ears from P3 mice were placed in ice-cold phosphate buffered saline (PBS), where the stria vascularis, the spiral ligament and the auditory nerve bundle were removed, leaving the organ of Corti. The tissue was then placed on 20 µL drops of polymerized rat tail collagen solution (Corning, NY, USA) in 35 mm tissue culture dishes and allowed to recover during 48 hours in DMEM:F12 (GE Healthcare Life Sciences, Hyclone Laboratories, Utah, USA) with added 1x N2 supplement, 25 mM Hepes, 1x non-essential aminoacids, 200 µg/mL ampicillin and 7% FBS, at 37°C and in 5% CO_2. Thereafter, the cultures were changed to fresh medium containing the corresponding AG. Preliminary experiments were conducted whereby cochlear explants were treated with GM at 0.1 mM, 0.2 mM and 2 mM concentrations, for 16 and 23 hours; treatment with 0.1 mM GM for 23 hours yielded OHC survival rates of approximately

50% and these were therefore the conditions selected for the evaluation of AG-induced toxicity on cochlear explants. Following AG treatment, the tissue was fixed for 2 hours in 4% paraformaldehyde and surviving HCs were identified by staining the actin filaments present in their stereocilia with FITC-conjugated phalloidin (Molecular Probes Inc., Life Technologies, Eugene, OR, USA) for 45 minutes, following a 5 minute-permeabilisation step in 3% Triton X-100 (Sigma-Aldrich, St. Louis, MO, USA) in PBS. Explants were mounted on microscope slides with Vectashield mounting medium containing diamidino-phenylindole staining (DAPI) (Vector laboratories, Burlingame, CA, USA); surviving HCs were imaged on a Nikon Eclipse 90i fluorescent microscope equipped with a DS-Ri1 digital CCD camera and counted all along the epithelium by using ImageJ software (Rasband, W.S., ImageJ, U. S. National Institutes of Health, Bethesda, Maryland, USA, http://imagej.nih.gov/ij/, 1997–2014). In each experiment, a set of explants were included that were not exposed to AG treatment and were therefore used as controls in order to establish the percentage of surviving HCs following AG exposure.

Confocal images were obtained with a Leica DMI 6000B microscope with TCS SP5 X confocal system and a WLL laser controlled by LAS AF software (Leica, Spain).

In vivo **ototoxicity analyses**

Functional CAP measurement and data collection procedures

Functional measurements were performed at five time points (before application, 60–90 minutes, day 7, 14, and 21). Measurements of compound action potentials (CAPs) were carried out under anaesthesia in a soundproof chamber and performed as described [1] (Supplementary material). For stimulus generation and recording of CAP and summation potential (SP) responses, a multi-function IO-Card (PCI-6052E or PCIe 6259, National Instruments, Austin, Texas, USA) was used. Sound stimulation was delivered to the ear in a calibrated free field system by a loudspeaker (Beyer DT 911, Beyer dynamic, Heilbronn, Germany), which was placed 3 cm distant from the animals' pinna. Sound pressure was calibrated with a ¼ inch B&K microphone probe system placed between the pinna and the

loudspeaker. For CAP threshold measurements, the stimulus with a cos^2-shaped tone pip of duration 3 ms and rise/fall times of 1 ms were presented. Silver wire electrodes (Ø 0.25mm, Goodfellow, Cambridgeshire, United Kingdom) were placed subcutaneously at the vertex (negative electrode), and beneath the dorsal neck musculature (ground electrode). Electrical signals were averaged by 24 repetitions of stimulus pairs with alternating phase. Responses were measured in the frequency range from 0.5 to 45.3 kHz, amplified by 74 dB and bandpass-filtered. For CAPs, responses were filtered between 0.2 and 5 kHz. The sound pressure level was increased in 1-dB step and limited to 120 dB SPL. An automated procedure was used to determine CAP thresholds [2]. CAP amplitude was defined as the difference in voltages between the first negative peak (N1) and the positive peak (P1), plotted against the current intensity. From the input-output (I/O) functions thus obtained, the values of CAP amplitude at 80 dB SPL were used to assess the relationship between functionality and histology as well as CAP threshold. For comparative analysis of functionality among AG and control groups, the CAP threshold and amplitude shifts were calculated as follows: threshold shifts were calculated by subtracting values before application from those at each time point. For CAP amplitude, the ratio between the value at day 21 and that before application was calculated and then used for analysis. To correlate the functional findings and histology, the frequency points from 0.5 to 45.3 kHz were subdivided into 2 frequency regions, based on the cochlear frequency map of the guinea pig, previously reported [3]. According to the report, the basal turn corresponds to a frequency of 4 to 45.3 kHz, and the 2nd turn to a frequency of 0.5 to 4 kHz. Therefore, frequency points from 0.5 to 4 kHz were defined as the 'Mid region', and those from 4 to 45.3 kHz as the 'Basal region'. CAP amplitude shifts at these frequency points were averaged for each region, and then the averaged value was defined as the CAP amplitude shift per ear.

SPs were measured in the frequency regions of 1, 1.4, 2, 2.8, 4, 5.6, 8, 11.3, 16, 22.6, and 32 kHz. SPs were evoked with 20 ms tone bursts and bandpass-filtered from 0.2 to 5 kHz. In controls, SPs were observed as a small shoulder preceding the CAPs. SP amplitude was

measured as the largest positive voltage occurring between 10 and 25 ms following stimulus onset, relative to the pre-stimulus baseline. CAP amplitude was defined as the amplitude of the first negative peak and the second positive peak between 5 and 10 ms for the SP/CAP ratio. The sound pressure levels for SPs were 0–100 dB SPL in steps of 5 dB, and CAP, SPs were plotted against current level to obtain I/O functions as described above. The SP/CAP amplitude ratios were calculated at 80 dB SPL using the values obtained at day 21 and those recorded before application, subdivided, and averaged into 2 regions, as described above. Regarding the SP/CAP amplitude ratio, the value before application was calculated in all groups, and used as a control.

Tissue preparation, immunohistochemistry, and hair cell and ribbon counts

Animals were euthanized 21 days after AG application in deep anaesthesia by an overdose of Narcoren (4 mL/kg). Then, the cochlear samples were collected, preserved, permeabilised, and blocked as described previously [4]. The cochlear tissues were micro-dissected for whole-mount processing, and then the obtained whole length of the organ of Corti was subdivided into 3 regions (basal, 2nd, and apical turn). As described above, the basal and 2nd turns correspond to the ‘Basal region’ and ‘Mid region’, respectively. Therefore, the two regions were used for staining. After the blocking procedure, the samples were incubated with primary antibodies in reaction buffer (0.5% normal donkey serum in 0.2% Triton-X100) overnight. The following primary antibodies were used: mouse-anti Myosin7A (Developmental Studies Hybridoma Bank; dilution 1:100), and rabbit-anti C-terminal-binding protein 2 (CtBP2, American research Products; dilution 1:250). After three PBS rinses, secondary antibodies were applied for 1 hour in reaction buffer. The following secondary antibodies were used: donkey-anti mouse Alexa 488 (Molecular Probes Inc., Life Technologies, Eugene, OR, USA; dilution 1:300), and goat-anti rabbit Cy3 (Jackson ImmunoResearch Laboratories; dilution 1:1500). Slices were mounted with DAPI-containing Vectashield mounting medium (Vector laboratories, Burlingame, CA, USA). Sections and whole-mount preparations were viewed using an Olympus BX61 microscope equipped with epifluorescence illumination or a Zeiss

Axio Imager 2 (Zeiss, Oberkochen, Germany) equipped with an Apotome.2 unit (Zeiss) using ZEN 2012 software (Zeiss).

For counting hair cells, the surviving outer hair cells (OHCs) were defined as DAPI- and Myosin7A-positive cells, and the surviving IHCs as Myosin7A-positive cells with CtBP2- and DAPI-positive nuclei. Ribbon counting was performed as described previously[5,6].

Supplementary Tables:

Supplementary Table S1. Activities of selected AGs against 61 clinical isolates.

Species	**Phenotype**	**Isolate (HUM-n)**	**Genta micin C1a**	**Genta micin**	**Paromo-mycin**	**Neo-mycin**	**Apra-mycin**
E. coli	ESBL	04-2283	64	>64	4	≤0.5	8
E. coli	ESBL	05-238	1	≤0.5	4	≤0.5	8
E. coli	ESBL	12.7	8	4	4	2	8
E. coli	ESBL	16.7	1	≤0.5	≤0.5	≤0.5	4
E. coli	ESBL	12.8	1	≤0.5	8	2	16
E. coli	ESBL	06.03	≤0,5	4	8	4	32
E. coli	ESBL	16.06	2	>64	4	2	8
E. coli	ESBL	24.11	1	>64	>64	>64	8
E. coli	ESBL	32.11	1	64	4	4	8
E. coli	ESBL	49.09	1	64	2	1	8
E. coli	AMP, FQ, SXT	14-2566	1	≤0.5	2	≤0.5	8
E. coli	AMP, FQ, SXT	14-2567	64	≤0.5	4	≤0.5	8
E. coli	AMP, FQ, SXT	14-3249	>64	2	>64	>64	16
E. coli	AMP, FQ, SXT	14-3702	1	≤0.5	2	≤0.5	8
E. coli	AMP, FQ, SXT	14-4412	1	≤0.5	2	≤0.5	4

K. pneumoniae	ESBL	8.14	64	≤0.5	2	≤0.5	8
K. pneumoniae	ESBL	11.11	>64	≤0.5	<0.5	≤0.5	4
K. pneumoniae	ESBL	12.17	16	>64	<0.5	≤0.5	2
K. pneumoniae	ESBL	6.10	8	≤0.5	4	≤0.5	8
K. pneumoniae	ESBL	11.03	>64	≤0.5	2	≤0.5	16
K. pneumoniae	ESBL	28.5	2	64	>64	32	4
K. pneumoniae	ESBL	25.3	>64	≤0.5	>64	8	4
K. pneumoniae	ESBL	6.19	>64	≤0.5	1	≤0,5	4
K. pneumoniae	ESBL	38.24	>64	64	4	2	>64
K. pneumoniae	ESBL	49.03	>64	32	>64	64	>64
K. pneumoniae	pABL (+)	282	1	32	2	2	4
K. pneumoniae	pABL (+)	284	>64	>64	4	4	8
K. pneumoniae	pABL (+)	286	>64	16	2	1	8
K. pneumoniae	pABL (+)	291	1	8	1	≤0.5	4
K. pneumoniae	pABL (+)	293	>64	32	1	1	4
A. baumannii	Carbapenem-R	17/AGL002	16	4	2	1	4
A. baumannii	Carbapenem-R	31/JUA002	8	16	>64	>64	8
A. baumannii	Carbapenem-R	59/RAC011	2	>64	4	4	16

A. baumannii	Carbapenem-R	133/SOR005	8	>64	>64	64	8
A. baumannii	Carbapenem-R	152/PUM009	>64	>64	>64	>64	32
A. baumannii	Carbapenem-S	5/CAR005	>64	≤0.5	1	≤0.5	4
A. baumannii	Carbapenem-S	7/LAL001	>64	>64	32	16	>64
A. baumannii	Carbapenem-S	12/SCQ004	>64	>64	>64	>64	16
A. baumannii	Carbapenem-S	16/AGL001	>64	≤0.5	1	1	4
A. baumannii	Carbapenem-S	19/CLI001	2	>64	32	8	16
P. aeruginosa	Carbapenem-R	12	2	16	4	1	2
P. aeruginosa	Carbapenem-R	19	>64	1	4	1	2
P. aeruginosa	Carbapenem-R	36	1	2	>64	8	8
P. aeruginosa	Carbapenem-R	60	≤0.5	8	>64	>64	64
P. aeruginosa	Carbapenem-R	238	>64	>64	>64	>64	64
P. aeruginosa	Carbapenem-R	81	>64	>64	>64	64	32
P. aeruginosa	Carbapenem-R	88	1	>64	16	2	8
P. aeruginosa	Carbapenem-S	22	≤0.5	>64	>64	8	32
P. aeruginosa	Carbapenem-S	90	≤0.5	>64	>64	64	64
P. aeruginosa	Carbapenem-S	75	1	2	>64	4	16
P. aeruginosa	Carbapenem-S	221	1	2	>64	8	16

S. aureus	*MRSA*<br>Linezolid-S	04-219	32	>64	>64	64	4
S. aureus	*MRSA*<br>Linezolid-S	04-47	>64	1	>64	>64	8
S. aureus	*MRSA*<br>Linezolid-R	07-1807	64	1	>64	>64	16
S. aureus	*MRSA*<br>Linezolid-R	09-6253	64	>64	>64	>64	16
S. aureus	*MRSA*<br>Linezolid-R	09-6297	32	>64	>64	>64	8
S. aureus	FQ, ERY	10-6939	64	<0.5	>128	>128	16
S. aureus	FQ, ERY	11-224	2	<0.5	<0.5	2	16
S. aureus	FQ, ERY	11-3395	4	2	1	<0.5	16
S. aureus	FQ, ERY	12-0175	>64	2	2	<0.5	16
S. aureus	FQ, ERY	14-1123	16	<0.5	2	<0.5	16

Supplementary Table S2. Different MICs of GM and GM C1a against 5 bacterial species of clinical isolates. MICs of GM C1a were at least 8 times higher than MICs of GM for 14 isolates (including 4/7 *P. aeruginosa* and 0/10 *S. aureus*), while the reverse was noted for 15 isolates (including 6/15 *K. pneumoniae*, 0/7 *P. aeruginosa* and 5/10 *S. aureus*).

	Number of isolates with MICs of	
	GM C1a ≥ 8 times higher than the MIC of GM	GM ≥ 8 times higher than the MIC of GM C1a
E. coli	4	2
K. pneumoniae	3	6
P. aeruginosa	4	0
A. baumannii	3	2
S. aureus	0	5
**Total**	14	15

Supplementary Table S3:hESI$^+$ probe parameters for the LCMS method

Spray voltage:	3500 V
Vaporiser temperature:	350 °C
Sheath gas pressure:	20
Ion sweep gas pressure:	0
Aux gas pressure:	10
Capillary temperature:	150 °C
Tube lens offset:	106
Skimmer offset:	0

Supplementary Figures:

Supplementary Figure S1

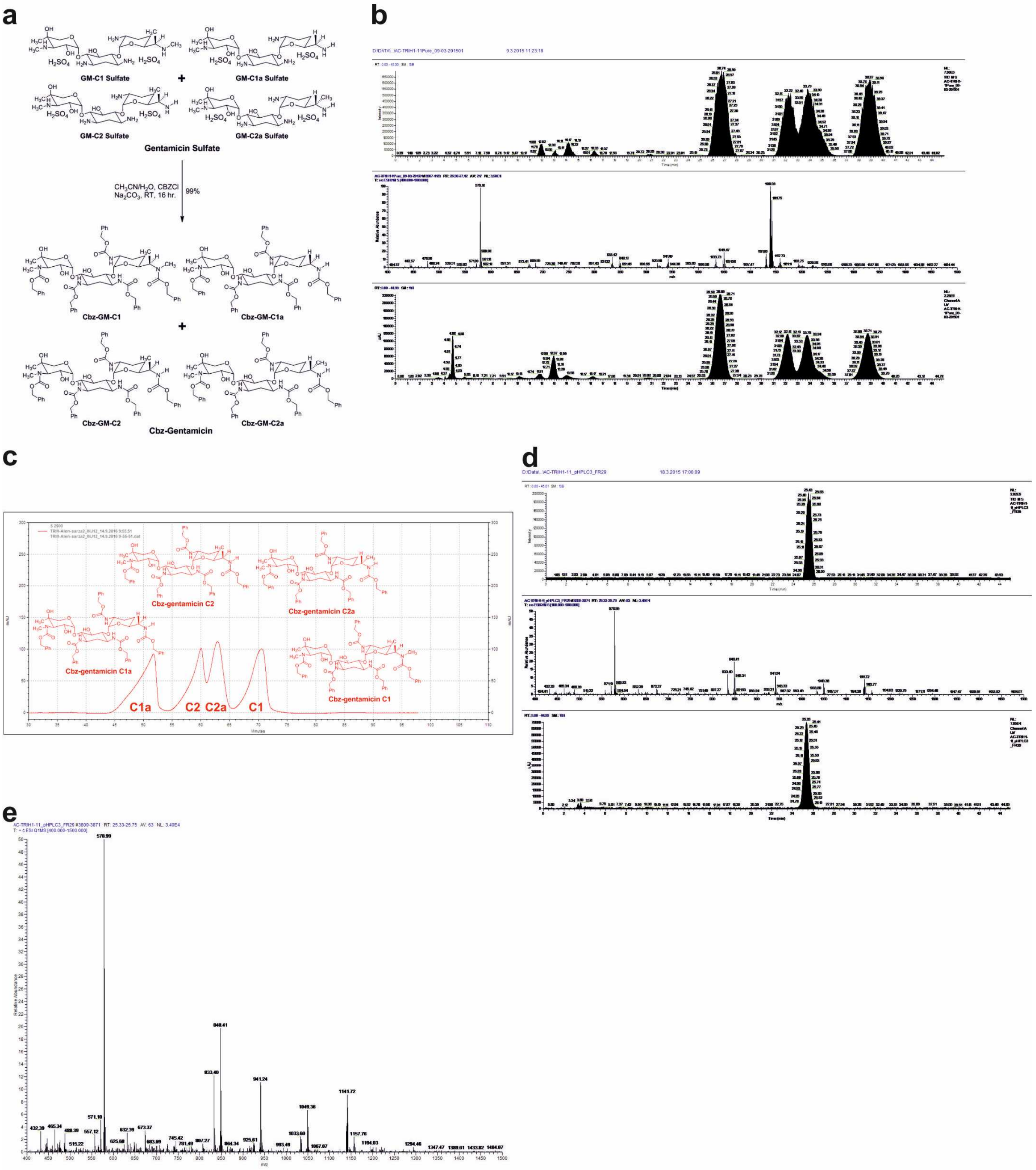

Supplementary Figure S1 (a) Derivatization of gentamicin C sulphate complex with benzyl chloroformate (Cbz-Cl). (b) Analytical HPLC-UV Chromatogram of Cbz-protected gentamicin congeners. Cbz-C1a congener (t_R = 26.68 min, 35.9 Area %), Cbz-C2 congener (t_R = 32.40 min, 19.05 Area %), Cbz-C2a congener (t_R = 34.06 min, 22.49 Area %) and Cbz-C1 congener (t_R = 39.01 min, 22.56 Area %). (c) Preparative HPLC-UV Chromatogram of Cbz-protected

gentamicin congeners. Loading capacity = 400 mg of GM C/injection. (d) LC-MS chromatogram of Cbz-protected gentamicin C1a purified on preparative HPLC. (e) Mass spectrum of Cbz-protected gentamicin C1a purified on preparative HPLC. MS $[\text{Cbz-C1a+ Na}]^+$ = 1141.73.

Supplementary Figure S2

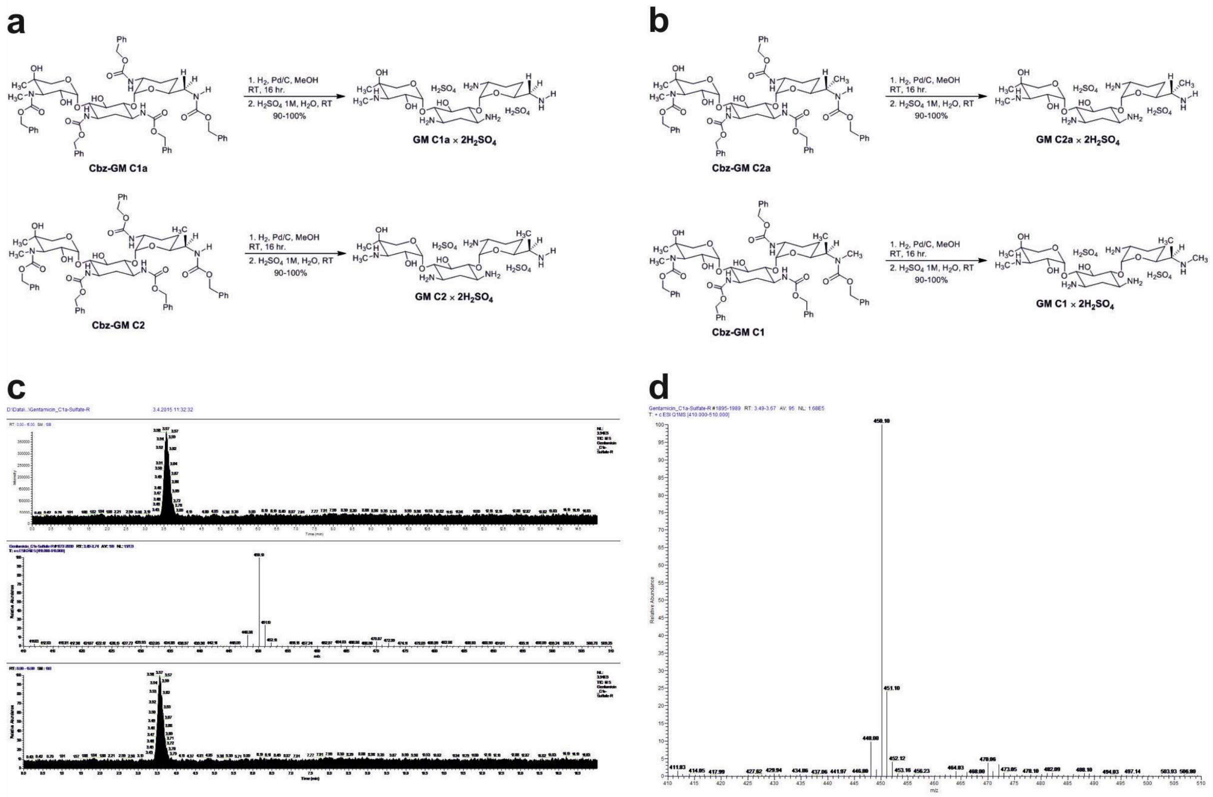

Supplementary Figure S2 (a) Deprotection of Cbz group from gentamicin C1a and C2 by Pd-catalyzed hydrogenation followed by preparation of disulfate salt. (b) Deprotection of Cbz group from gentamicin C2a and C1 by Pd-catalyzed hydrogenation followed by preparation of disulfate salt. (c) LC-MS chromatogram of gentamicin C1a after hydrogenation and sulfate preparation. (d) Mass spectrum of gentamicin C1a after hydrogenation and sulfate preparation. MS $[C1a + H]^+$ = 450.10.

Supplementary Figure S3

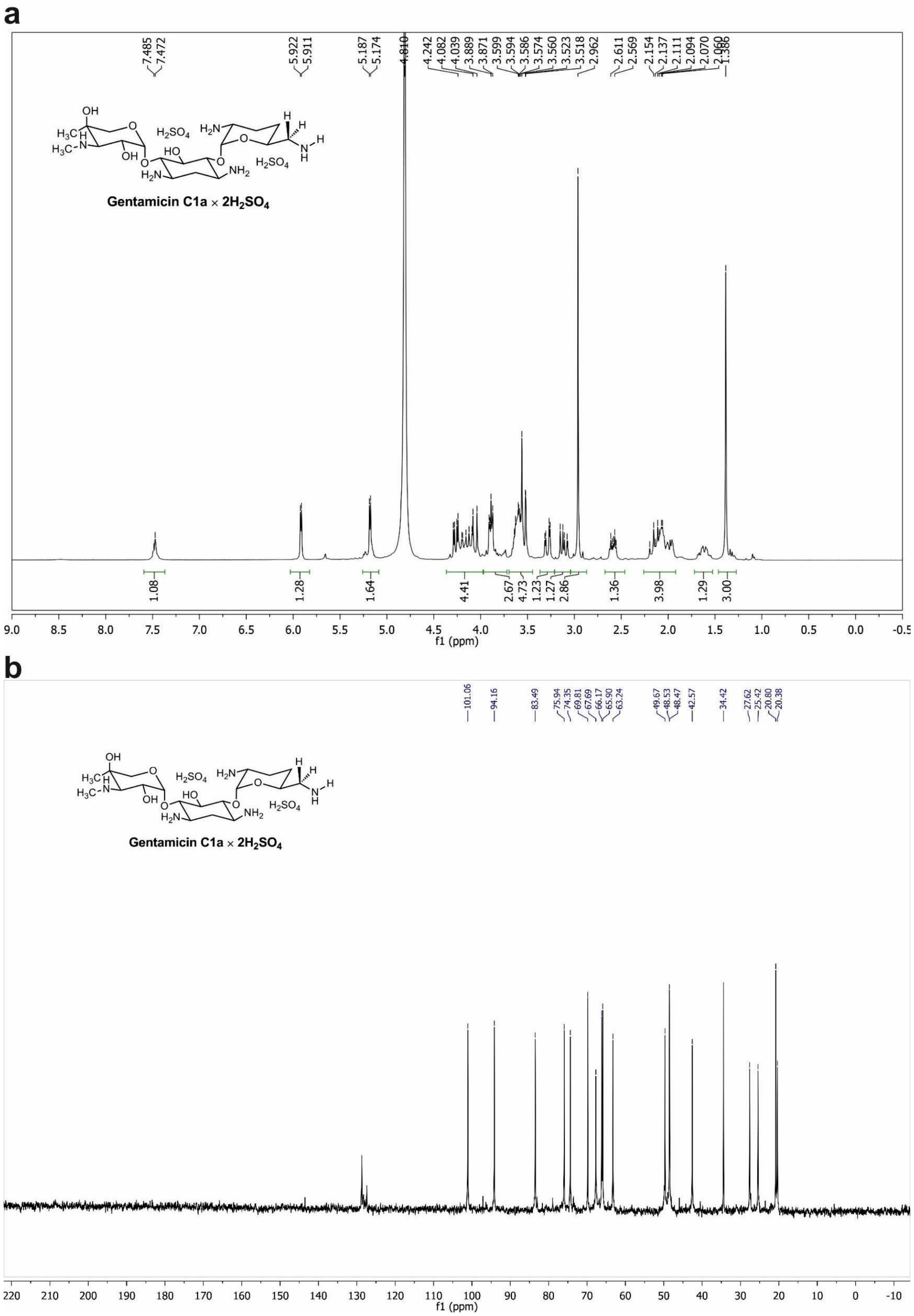

Supplementary Figure S3 (a) ^{1}H-NMR spectrum of gentamicin-C1a recorded at 300 MHz NMR spectrometer in D_2O. (b) ^{13}C-NMR spectrum of gentamicin-C1a recorded at 300 MHz

NMR spectrometer in D_2O. ^1H- and ^{13}C-NMR spectra were recorded on Agilent Technologies Unity Inova on a 300 MHz NMR spectrometer in deuterium oxide (D_2O) at ambient temperature. Chemical shifts (δ) are given in ppm and calibrated using the signal of residual solvent (D_2O: δ_H = 4.80 ppm).

Gentamicin-C1a disulphate

^{1}H NMR (300 MHz, D_2O) δ 7.48-7.46 (m, 1H), 5.91 (d, *J* = 3.5 Hz, 1H), 5.17 (d, *J* = 3.7 Hz, 2H), 4.32-4.03 (m, 4H), 3.93-3.81 (m, 3H), 3.68-3.51 (m, 5H), 3.28 (dd, *J* = 13.4, 3.0 Hz, 1H), 3.10 (dd, *J* = 13.4, 8.5, Hz, 1H), 2.95 (s, 3H), 2.57 (dt, *J* = 8.4, 4.2 Hz, 1H), 2.19-1.94 (m, 4H), 1.60 (qd, *J* = 11.8, 4.8 Hz, 1H), 1.38 (s, 3H) ppm. **^{13}C NMR** (75 MHz, D_2O) δ 101.06, 94.16, 83.49, 75.94, 74.35, 69.81, 67.69, 66.17, 65.90, 63.24, 49.67, 48.53, 48.47, 42.57, 34.42, 27.62, 25.42, 20.80, 20.38 ppm. **MS** (ESI, $[M + H]^+$) calculated for $C_{19}H_{40}N_5O_7$ 450.29 found 450.10

Supplementary Figure S4

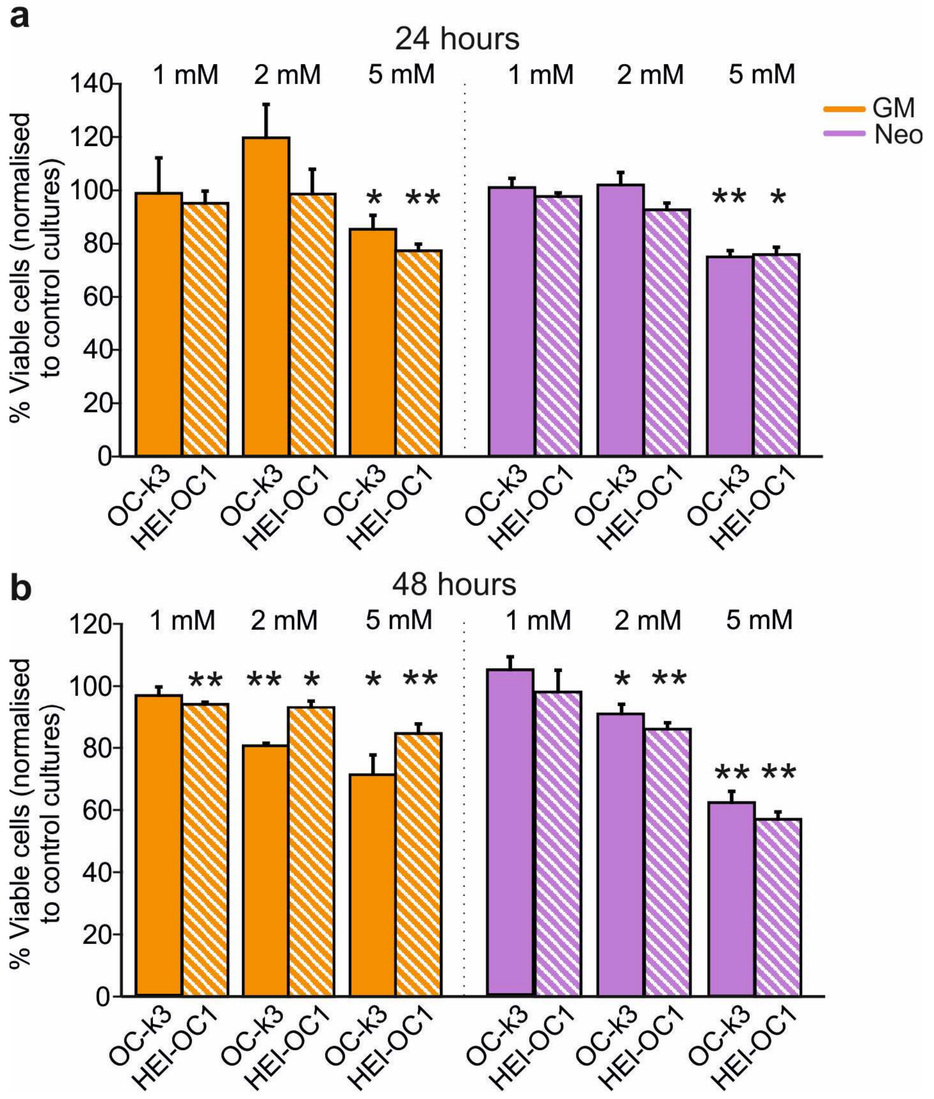

Supplementary Figure S4. Viability of OC-k3 and HEI-OC1 cell cultures following treatment with the AGs GM and neomycin.

Viability of the otic cell cultures OC-k3 (solid bars) and HEI-OC1 (striped bars) following 24-hour (a) and 48-hour treatments (b) with various concentrations (1, 2 and 5 mM) of the AGs GM and Neo, as measured by the MTT viability test and compared to control cultures (% viable cells in controls=100%). Data are presented as the mean ± SD (two-tailed unpaired Student´s t-tests were used to compare each treatment to controls; *p<0,05; **p<0,01; (a) 24-hour treatments on OC-k3 cells: 1 mM GM, $p = 0.93$; 2 mM GM, $p = 0.26$; 5 mM GM, $p = 0.04$; 1 mM Neo, $p = 0.82$; 2 mM Neo, $p = 0.73$; 5 mM Neo, $p = 0.008$; (a) 24-hour treatments on HEI-OC1 cells: 1 mM GM, $p = 0.39$; 2 mM GM, $p = 0.88$; 5 mM GM, $p = 0.0002$; 1 mM Neo, p

= 0.2; 2 mM Neo, $p = 0.1$; 5 mM Neo, $p = 0.01$; (b) 48-hour treatments on OC-k3 cells: 1 mM GM, $p = 0.3$; 2 mM GM, $p = 0.0002$; 5 mM GM, $p = 0.046$; 1mM Neo, $p = 0.38$; 2 mM Neo, $p = 0.03$; 5 mM Neo, $p = 0.009$; (b) 48-hour treatments on HEI-OC1 cells: 1 mM GM, $p = 0.003$; 2 mM GM, $p = 0.046$; 5 mM GM, $p = 0.007$; 1 mM Neo, $p = 0.81$; 2 mM Neo, $p = 0.0005$; 5 mM Neo, $p = 0.003$). Abbreviations: AGs, aminoglycosides; GM, gentamicin; MTT, 3-[4,5-dimethylthiazol-2-yl]-2,5-diphenyltetrazolium bromide; Neo, neomycin.

Supplementary Figure S5

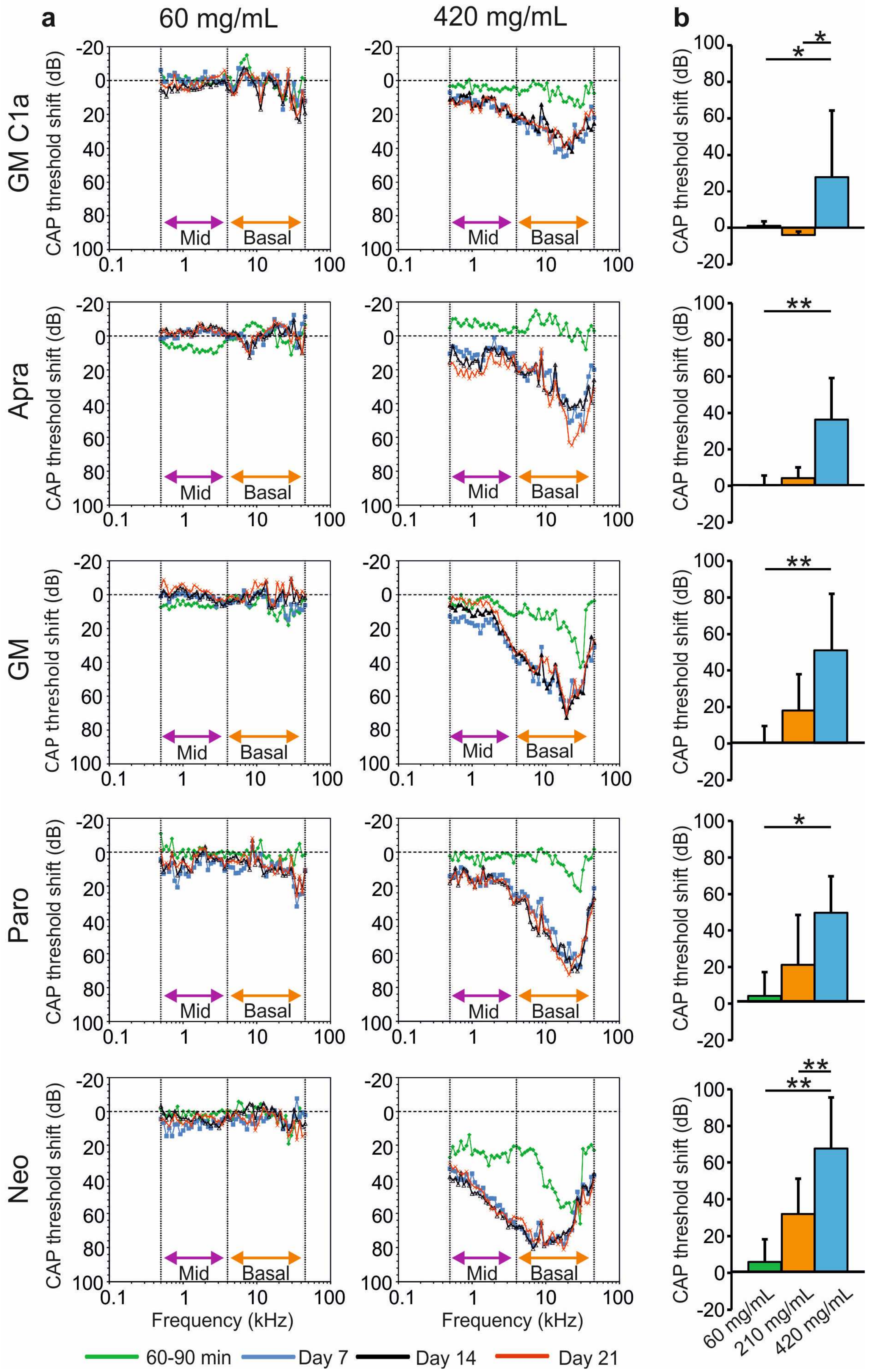

1

Supplementary Figure S5. Time course and dose-dependent effects of AGs on CAP threshold shifts.

(a) Time course of CAP threshold shifts at 4 different time points from 0.5 to 45.3 kHz following administration of 60 and 420 mg/mL of 5 selected AGs. The points are mean values. (b) Comparison of CAP threshold shifts at the basal region on day 21 and dose-dependent effect of each AG. Data are presented as the mean ± SD (One-way ANOVA with Tukey's multiple comparison test, $*p<0.05$; $**p<0.01$; $***p<0.001$ (b) GM C1a $p = 0.04$, Apra $p = 0.001$, GM $p = 0.0053$, Paro $p = 0.0163$, Neo $p = 0.0005$). Abbreviations: Apra, apramycin; GM, gentamicin; GM C1a, gentamicin C1a; Neo, neomycin, Paro, paromomycin. (n = 3 animals, 6 ears per concentration).

References

1 Muller, M., Tisch, M., Maier, H. & Lowenheim, H. Reduction of permanent hearing loss by local glucocorticoid application : Guinea pigs with acute acoustic trauma. *Hno* **65**, 59-67, doi:10.1007/s00106-016-0266-z (2017).

2 Muller, M., Smolders, J. W., Ding-Pfennigdorff, D. & Klinke, R. Regeneration after tall hair cell damage following severe acoustic trauma in adult pigeons: correlation between cochlear morphology, compound action potential responses and single fiber properties in single animals. *Hearing research* **102**, 133-154 (1996).

3 Tsuji, J. & Liberman, M. C. Intracellular labeling of auditory nerve fibers in guinea pig: central and peripheral projections. *J Comp Neurol* **381**, 188-202 (1997).

4 Bako, P. *et al.* Methyl methacrylate embedding to study the morphology and immunohistochemistry of adult guinea pig and mouse cochleae. *J Neurosci Methods* **254**, 86-93, doi:10.1016/j.jneumeth.2015.07.017 (2015).

5 Chumak, T. *et al.* BDNF in Lower Brain Parts Modifies Auditory Fiber Activity to Gain Fidelity but Increases the Risk for Generation of Central Noise After Injury. *Molecular neurobiology* **53**, 5607-5627, doi:10.1007/s12035-015-9474-x (2016).

6 Zuccotti, A. *et al.* Lack of brain-derived neurotrophic factor hampers inner hair cell synapse physiology, but protects against noise-induced hearing loss. *J Neurosci* **32**, 8545-8553, doi:10.1523/JNEUROSCI.1247-12.2012 (2012).
